# Can we abandon phosphorus starter fertilizer in maize? Results from a diverse panel of elite and doubled haploid landrace lines of maize (*Zea mays* L.)

**DOI:** 10.3389/fpls.2022.1005931

**Published:** 2022-12-16

**Authors:** Sandra Roller, Thea M. Weiß, Dongdong Li, Wenxin Liu, Wolfgang Schipprack, Albrecht E. Melchinger, Volker Hahn, Willmar L. Leiser, Tobias Würschum

**Affiliations:** ^1^ Institute of Plant Breeding, Seed Science and Population Genetics, University of Hohenheim, Stuttgart, Germany; ^2^ State Plant Breeding Institute, University of Hohenheim, Stuttgart, Germany; ^3^ Key Laboratory of Crop Heterosis and Utilization, The Ministry of Education, Key Laboratory of Crop Genetic Improvement, Beijing Municipality, National Maize Improvement Center, College of Agronomy and Biotechnology, China Agricultural University, Beijing, China

**Keywords:** maize, phosphorus, starter fertilization, landraces, genetic variation

## Abstract

The importance of phosphorus (P) in agriculture contrasts with the negative environmental impact and the limited resources worldwide. Reducing P fertilizer application by utilizing more efficient genotypes is a promising way to address these issues. To approach this, a large panel of maize (*Zea mays* L.) comprising each 100 Flint and Dent elite lines and 199 doubled haploid lines from six landraces was assessed in multi-environment field trials with and without the application of P starter fertilizer. The treatment comparison showed that omitting the starter fertilizer can significantly affect traits in early plant development but had no effect on grain yield. Young maize plants provided with additional P showed an increased biomass, faster growth and superior vigor, which, however, was only the case under environmental conditions considered stressful for maize cultivation. Importantly, though the genotype-by-treatment interaction variance was comparably small, there is genotypic variation for this response that can be utilized in breeding. The comparison of elite and doubled haploid landrace lines revealed a superior agronomic performance of elite material but also potentially valuable variation for early traits in the landrace doubled haploid lines. In conclusion, our results illustrate that breeding for P efficient maize cultivars is possible towards a reduction of P fertilizer in a more sustainable agriculture.

## 1 Introduction

In 2018, Germany was convicted by the European Court of Justice for inadequate implementation of the EU Nitrates Directive (Council of the European Union, 1991). The EU Commission had sued because of, among other issues, the detrimental ecological condition of the coastal waters due to eutrophication by phosphorus (P) (EuGH, 2018). The pending lawsuit and the opposing positions from stakeholders led to a public discussion around the necessity, the environmental consequences and the prospect of higher efficiency use of P fertilizer.

Phosphorus is one of the most crucial fertilizer components, with over 40 million tons used worldwide, 90% thereof in agriculture (Killiches et al., 2013). P is vital for high-input agriculture as it is an essential plant nutrient and not replaceable. The assimilated P is incorporated into nucleic acids, phospholipids and enzymes, it is a component of intracellular energy transfer, maintains membrane structures and is crucial for photosynthesis (White and Hammond, 2008; Ahemad et al., 2009). However, the regular fertilization has led to an oversaturated soil P level in many regions of Europe (Tóth et al., 2014; Ballabio et al., 2019). In such areas, suspension of P fertilization over several years had little effect on the yield of various crops (Römer, 2009; von Tucher et al., 2017). Despite this, with a P supply of more than 20 mg P_2_O_5_ per 100 g of soil after the calcium acetate lactate (CAL) method, the official recommended maximum level of P fertilization still corresponds to the amount of P removal (Klages and Schultheiß, 2020). P cannot be substituted and only a small proportion of its limited reserves are readily available. Various forecasts estimate that a “Peak Phosphorus”, the timepoint where supply will no longer be able to keep up with demand, could ensue in a period between 30 - 300 years (Cordell and White, 2011). Acute price rises such as in 2008 by 800% (Cordell and White, 2011) for phosphate rock and by approximately 76% in March 2022 for Diammonium phosphate compared to the previous year, provoked by growing input costs, supply disruptions and export restrictions, increase concerns around P fertilizer availability and affordability (World Bank, 2022). Consequently, a reduced P fertilization and resulting lower P levels in the soil should be contemplated.

A substantial reduction of fertilizer usage in agriculture would require genotypes to be more phosphate-use-efficient (PUE), which would include, on the one hand, the enhanced uptake and, on the other hand, the improved allocation of P in biomass growth (Schröder et al., 2011; Veneklaas et al., 2012). Genotypes being PUE would provide the same yield even under reduced P soil concentration and PUE is, therefore, a desired goal in breeding. Performance comparisons between doubled haploid landrace lines and modern elite lines revealed a similar or even a reduced PUE in modern lines (Li et al., 2021). A prevailing hypothesis for this putative trend is that landraces have an enriched gene pool with beneficial alleles, which may have been lost during the breeding process of modern varieties under optimal fertilized conditions (Bellon, 2009; Wissuwa et al., 2009).

Application of P fertilizer can increase the yield of crops like maize (*Zea Mays* L.) up to 65% under low P soil conditions (Onasanya et al., 2009; Amanullah et al., 2010). Maize is one of the most important crops for global food security next to wheat (*Triticum aestivum* L.) and rice (*Oryza sativa* L.) (FAOSTAT, 2021). In Germany, land cultivated with maize showed a steady increase over the last 60 years. In 2020, maize was grown on over 2.7 million ha and continues to gain importance mainly as animal feed and energy maize (Statistisches Bundesamt, 2019). Additional P fertilization in the form of starter fertilizer provided to the plants at sowing has become established in practical farming as maize has a high nutrient requirement (Barry and Miller, 1989; Renu et al., 2015) and a poor ability to absorb P during the early developmental stages (Nadeem et al., 2013).

The environmental strain and the limited reserves of P inevitably result in the responsibility of dealing more rationally with P in agriculture, where the primary usage lies. In this context, the question arises as to whether the current practice of P fertilization is still reasonable or if not a reduction of P application is feasible with appropriate PUE varieties. We approached this question with a diverse panel of maize genotypes, including both Flint and Dent elite lines that represent the heterotic pattern prevalent in Central Europe and at the same time two populations that have been separated for centuries. In addition, doubled haploid lines from six European Flint landraces were also investigated in this study to test the hypothesis that selection of elite material mainly under optimal agricultural conditions eroded the need for specialized adaptions, such as traits for efficient phosphorus acquisition (Li et al., 2021). The doubled haploid lines from the landraces allow the preservation of the genetic variation in only one step from a heterogenous population and facilitate replicated trials (Strigens et al., 2013) to examine the potential of doubled haploid lines under varying P levels in this study.

Past studies on P stress response mechanisms often applied severe P deficiency to screen the genotypes (Yun and Kaeppler, 2001; Zhu et al., 2005). However, whether the so identified P stress tolerance mechanisms are also beneficial for the plants under moderate stress conditions is unclear. The fields used for this study therefore represent the typical status of moderate to high P soil content in Central Europe (Tóth et al., 2014; Ballabio et al., 2019) and thus allow to investigate the effects of the omission of P starter fertilizer on maize performance in a realistic setting. In particular, we examined plant development and yield in a multi-environment field trial to i) dissect the phenotypic response of maize with and without P starter fertilization, ii) characterize and compare the phenotypic response of Dent and Flint elite lines as well as landrace doubled haploid lines, and iii) evaluate the potential for future varieties adapted to a lower P application.

## 2 Material and methods

### 2.1 Plant material

For this study, a large panel with 399 genotypes comprising 100 Flint and 100 Dent elite lines and 199 doubled haploid lines from landraces was used. The Flint and Dent lines, generated by recurrent selfing or doubled haploid technology, stem from the maize breeding program of the University of Hohenheim. The landrace lines are doubled haploid lines produced from six European Flint landraces, namely Campan Galade (CAMP; n = 11), Gelber Badischer Landmais (GELB; n = 32), Satu Mare (SATU; n = 53), St Galler Rheintaler (STGA; n = 14), Strenzfelder (STRE; n = 30) and Walliser (WALL; n = 59) and are a subset of larger panel described in a previous study (Böhm et al., 2017). Due to heterogeneity or lack of cob formation, 53 genotypes (51 landrace doubled haploid lines and two Dent lines) from a initially larger panel of 450 lines were excluded from the field trials after the first year. The two Dent lines were replaced, resulting in a total of 399 genotypes used in this study.

### 2.2 Field trials

The field trials were performed in three different environments (location × year combinations), i.e., Hohenheim (HOH) in the years 2019 (HOH19) and 2020 (HOH20), as well as Eckartsweier in 2020 (EWE20). Specific characteristics of the environments and soil properties before the field trials are given in **Table 1**. The weather data for both locations and years are depicted in **Figure S1**.

**Table 1:** Properties of the environments.

Environment	Altitude [m]	Ø Temp [°C]	Ø P recip [mm]	P_2_O_5_ [mg/100 g soil]	P availability classification	pH	Growing season
HOH19	400	15.15	80.36	19.50	D	6.79	30.04 – 24.10
HOH20	400	15.26	57.27	14.40	D	6.95	20.04 – 13.10
EWE20	140	16.49	56.90	9.90	C	5.66	15.04 – 16.09

Description of environments showing the altitude over sea level, the average temperature [°C] and average precipitation [mm] during the field season, soil P content [mg/100 g] (CAL method) and classification, pH value and sowing/harvest date. P classification according to VDLUFA-P-content-classes (A = very low, E = very high) defined by the Association of German Agricultural Analytic and Research Institutes (Verband Deutscher Landwirtschaftlicher Untersuchungs- und Forschungsanstalten). Weather data was taken from www.wetter-bw.de for the weather stations Hohenheim (AGM 103) and Eckartsweier (AGM 1).

Field trials were designed as an alpha lattice 90 × 5 design in HOH19 and an alpha lattice 80 × 5 design in the other environments, with two replications for each genotype resulting in a total of 1600 plots (1800 in HOH19). The entries were planted in two-row plots of 4 m length, inter-row spacing of 0.75 m, and allies of 0.8 m width resulting in a net plot size of 6 m^2^ with a sowing density of 8.66 plants/m^2^. A control (-P) and a starter fertilizer treatment (+P) were realized. The starter fertilizer contained 115 kg Triple Superphosphate (TSP) per ha, resulting in 52.9 kg P/ha. The control treatment was not provided with additional P starter fertilizer. Plant protection with herbicides and *Trichogramma* was done according to the field situation and local practices.

### 2.3 Trait scoring and statistical analysis

Eleven traits related to growth, development and yield were assessed. Plant height (PH) [cm] was determined two to four times (PH1-4) during the growing season as the average value of three plants in each plot measured from the ground to the tip of the longest leaf. For final plant height (PHF) [cm] the distance from the ground to the tassel was measured on three plants and averaged while in EWE20 PHF was assessed on a plot basis. Early vigor (EV) was determined by visual scoring on plot basis adopting a scale from one (poor) to nine (excellent) (Peter et al., 2009). Visual evaluation of purpleness (PR) was done on plot basis from one (no coloration) to three (dark purple) in HOH20 (Presterl et al., 2007). Early Biomass (BM) [g] was assessed as the average weight of dry biomass (at BBCH stage ~ V4-V6) of four plants. The anthesis-silking interval (ASI) [days] was calculated by the difference between days to silking (50% visible silks per plot) and days to anthesis (DTA) (50% anthesis per plot) (Edmeades et al., 2000). Grain yield (GY) [t/ha] is based on the yield of grain per plot corrected for its moisture content. The average grain dry matter content (GDM) [%] at harvest was determined in relation to the fresh weight after drying for 72 h at 110°C.

The phenotypic data was subjected to the Bonferroni-Holm outlier detection (Bernal-Vasquez et al., 2016) with the R-package ‘multtest’ (Pollard et al., 2005). Out of the 450 lines in HOH19, 53 were not considered in the further analyses but were included in the estimation of variance components as a separate group with dummy variables to maintain the block effect. Dummy variables were introduced for each separate group to estimate population-specific variance components. The variance components were estimated by treating all factors, except general mean and P treatment, as random.

All subsequent analyses were performed with the best linear unbiased estimates (BLUEs) obtained by taking the factor genotype as fixed. The statistical model on a single environment level was:

*y_ijk_ = μ + g_i_ + r_j_ + b_jk_ + ϵ_ijk_*

where *y_ijk_* is the trait value of the i-th genotype in the k-th block nested within the j-th replicate, *µ* the overall mean, *g_i_* the effect of i-th genotype, *r_j_* the effect of j-th replicate, *b_jk_* the effect of the k-th block nested within the j-th replicate, and *ϵ_ijk_* the residual error effect. To estimate the genotype-by-treatment interaction variance, a model across the two P treatments was used, in which the above model was extended by an effect for the P treatment and the genotype-by-treatment interaction. Heritability was estimated according to Hallauer et al. (2010):


$$H^{2}=\;\frac{\sigma_{G}^{2}}{\sigma_{G}^{2}+\;\frac{\sigma_{g\times e}^{2}}{n_{e}}+\;\frac{\sigma_{e}^{2}}{n_{e}*\;n_{r}}}$$

where $\sigma_{G}^{2}$ stands for the genotypic variance, $\sigma_{g\times e}^{2}$ for the genotype-by-environment interaction variance, $\sigma_{e}^{2}$ denotes the error variance, *n*_*e*_ the number of environments and *n*_*r*_ the number of replications.

Statistical analyses were performed with RStudio 4.1.2 (RStudio Team, 2020). All mixed model calculations were done with ASRemL-R 3.0 (Butler et al., 2009). Comparisons of treatments, environments and populations were made with an analysis of variance (ANOVA) with the R package ‘agricolae’ (de Mendiburu, 2021). The packages ‘ggplot2’ (Wickham, 2016), ‘fmsb’ (Nakazawa, 2022) and ‘ggfortify’ (Tang et al., 2016) were used to produce the plots.

## 3 Results

### 3.1 Summary statistics under two P fertilizer treatments

Heritabilities were generally high, ranging from 0.69 for biomass in HOH19 to 0.98 for days to anthesis in HOH20 (**Table 2**). Heritabilities were similar between the two P treatments for all traits and we observed a significant genotypic variance ( $\sigma_{G}^{2}$ ) for all traits in both treatments. The genotype-by-treatment interaction variance ( $\sigma_{G\times T}^{2}$ ) was significant for most traits but considerably smaller than the genotypic variance across treatments. In addition, for the plant height measurements, the three values per plot allowed to estimate the intra-plot variation, which for HOH19 and HOH20 was higher in the -P than in the +P treatment.

**Table 2:** Summary statistics for the evaluated traits.

-P							Across Treatment			+P						
	min	mean ± sd	max	$\sigma_{G}^{2}$	$\sigma_{e}^{2}$	H^2^	$\sigma_{G\times T}^{2}$	$\sigma_{G}^{2}$	r	*H^2^*	$\sigma_{e}^{2}$	$\sigma_{G}^{2}$	min	mean ± sd	max	
Plant Height 1 (PH1)										Plant Height 1 (PH1)						
HOH19	16.17	29.72 ± 5.87a	50.25	29.19^***^	8.12	0.88	2.00^***^	39.01^***^	0.87	0.92	8.63	52.53^***^	10.79	34.69 ± 7.64b	60.62	HOH19
HOH20	12.82	22.18 ± 4.14c	35.17	14.68^***^	4.06	0.88	0.68^**^	16.98^***^	0.83	0.87	6.30	20.96^***^	12.95	24.02 ± 4.98d	40.08	HOH20
EWE20	13.47	19.15 ± 2.36e	26.17	4.57^***^	1.75	0.84	0.12^ns^	4.77^***^	0.80	0.85	1.87	5.25^***^	12.85	19.32 ± 2.53e	25.99	EWE20
Early Vigor (EV)										Early Vigor (EV)						
HOH19	0.95	4.66 ± 1.07a	7.18	0.90^***^	0.35	0.84	0.11^***^	1.04^***^	0.75	0.85	0.51	1.41^***^	1.13	5.75 ± 1.32b	9.20	HOH19
HOH20	1.46	4.46 ± 1.21a	7.29	1.30^***^	0.25	0.91	0.03^*^	1.26^***^	0.86	0.90	0.30	1.28^***^	2.04	5.41 ± 1.21c	8.05	HOH20
EWE20	1.97	7.12 ± 0.93d	9.21	0.77^***^	0.15	0.91	0.04^***^	0.72^***^	0.81	0.85	0.26	0.75^***^	2.95	6.77 ± 0.96e	9.09	EWE20
Biomass (BM)										Biomass (BM)						
HOH19	1.36	4.98 ± 1.79a	11.52	2.00^***^	1.82	0.69	0.69^***^	3.19^***^	0.62	0.82	2.51	5.88^***^	1.47	7.68 ± 2.82b	18.53	HOH19
HOH20	0.73	3.34 ± 1.19c	7.66	1.14^***^	0.43	0.84	0.13^***^	1.53^***^	0.77	0.82	0.98	2.22^***^	0.77	4.14 ± 1.68d	9.82	HOH20
EWE20	1.88	11.02 ± 2.99e	26.26	7.06^***^	2.45	0.85	0.45^**^	5.84^***^	0.70	0.79	3.05	5.66^***^	4.23	10.61 ± 2.80e	19.54	EWE20
Purpleness (PR)										Purpleness (PR)						
HOH20	1.00	2.00 ± 0.63a	3.00	0.35^***^	0.11	0.86	0.04^***^	0.26^***^	0.73	0.80	0.13	0.24^***^	0.96	1.64 ± 0.56b	3.02	HOH20
Days to Anthesis (DTA)										Days to Anthesis (DTA)						
HOH19	78.48	88.58 ± 4.41a	106.00	18.93^***^	0.99	0.97	0.55^***^	16.22^***^	0.95	0.97	0.81	14.14^***^	78.00	87.34 ± 3.82b	103.00	HOH19
HOH20	78.80	94.22 ± 5.33c	112.12	27.69^***^	1.12	0.98	0.98^***^	24.19^***^	0.93	0.97	1.46	22.90^***^	78.26	94.10 ± 4.87c	109.94	HOH20
EWE20	72.7	84.04 ± 4.67d	101.1	20.87^***^	1.58	0.96	0.37^***^	20.31^***^	0.94	0.97	1.42	20.61^***^	72.4	84.79 ± 4.63d	101.90	EWE20
Grain yield (GY)										Grain yield (GY)						
HOH19	0.11	2.47 ± 1.76a	7.69	2.96^***^	0.22	0.96	0.10^***^	2.81^***^	0.93	0.97	0.16	2.83^***^	0.05	2.40 ± 1.71ab	6.74	HOH19
HOH20	-0.03	0.93 ± 0.79c	3.24	0.57^***^	0.09	0.93	0.01^**^	0.63^***^	0.92	0.95	0.08	0.70^***^	-0.01	1.04 ± 0.87c	3.71	HOH20
EWE20	-0.02	2.14 ± 1.49b	6.37	2.13^***^	0.19	0.96	0.01^ns^	2.15^***^	0.96	0.97	0.14	2.14^***^	0.04	2.31 ± 1.49ab	6.43	EWE20
Anthesis - Silking Interval (ASI)										Anthesis - Silking Interval (ASI)						
HOH19	-3.44	2.91 ± 2.63a	13.52	6.01^***^	1.67	0.88	0.23^**^	5.92^***^	0.85	0.89	1.48	6.27^***^	-1.93	3.32 ± 2.67a	14.30	HOH19
HOH20	-0.09	5.23 ± 3.42b	17.91	10.16^***^	2.57	0.89	0.62^***^	9.27^***^	0.82	0.90	1.86	8.81^***^	-2.00	4.31 ± 3.14c	21.00	HOH20
EWE20	-6.96	1.37 ± 2.25d	10.06	3.96^***^	1.91	0.81	0.08^ns^	4.05^***^	0.80	0.84	1.59	4.26^***^	-9.04	1.32 ± 2.29d	11.50	EWE20
Grain dry matter (GDM)										Grain dry matter (GDM)						
HOH19	31.88	69.88 ± 5.56a	100.59	19.98^***^	14.44	0.74	< 0.01^ns^	21.75^***^	0.76	0.72	14.52	18.35^***^	38.43	68.10 ± 5.44b	100.00	HOH19
HOH20	34.69	71.63 ± 7.35c	82.81	47.19^***^	9.81	0.91	2.09^***^	36.29^***^	0.83	0.92	4.95	28.32^***^	42.46	73.67 ± 5.87d	83.48	HOH20
EWE20	59.77	79.22 ± 5.04e	87.07	23.83^***^	2.41	0.95	0.46^***^	21.70^***^	0.92	0.96	1.73	20.71^***^	63.55	82.07 ± 4.65f	88.85	EWE20
Plant Height 2 (PH2)										Plant Height 2 (PH2)						
HOH19	19.85	70.84 ± 12.78a	108.99	133.06^***^	25.02	0.91	6.32^***^	134.00^***^	0.85	0.91	27.81	139.31^***^	21.12	76.89 ± 12.99b	119.61	HOH19
HOH20	14.21	26.56 ± 5.49c	42.56	27.44^***^	4.46	0.93	1.15^***^	24.86^***^	0.85	0.88	6.64	24.89^***^	16.05	28.26 ± 5.36cd	43.88	HOH20
EWE20	15.80	28.11 ± 3.90c	39.53	12.65^***^	4.10	0.86	0.82^**^	15.09^***^	0.83	0.89	4.99	19.41^***^	16.71	29.86 ± 4.78d	43.90	EWE20
Plant Height Final (PHF)										Plant Height Final (PHF)						
HOH19	93.08	154.35 ± 20.14a	211.97	367.32^***^	65.02	0.92	4.39^ns^	352.52^***^	0.90	0.93	54.26	347.85^***^	86.18	153.25 ± 19.64a	216.83	HOH19
HOH20	58.04	119.95 ± 18.57b	164.83	308.03^***^	56.00	0.92	5.50^ns^	301.20^***^	0.88	0.91	61.71	305.03^***^	56.87	121.45 ± 18.55b	166.35	HOH20
EWE20	91.92	168.09 ± 22.01c	228.08	446.88^***^	58.04	0.94	5.99^ns^	417.97^***^	0.91	0.93	64.67	400.59^***^	97.90	165.24 ± 20.95c	224.32	EWE20
Plant Height 3 (PH3)										Plant Height 3 (PH3)						
HOH20	14.02	34.60 ± 7.50a	53.53	50.11^***^	10.06	0.91	2.42^***^	42.58^***^	0.83	0.89	10.01	40.04^***^	19.21	37.13 ± 6.81b	56.16	HOH20
EWE20	20.70	47.91 ± 7.35c	68.05	47.54^***^	10.03	0.91	2.82^***^	42.75^***^	0.82	0.91	8.87	44.90^***^	27.81	47.92 ± 7.22c	69.28	EWE20
Plant Height 4 (PH4)										Plant Height 4 (PH4)						
HOH20	29.90	80.20 ± 15.67a	126.20	220.78^***^	38.51	0.92	4.56^*^	198.34^***^	0.87	0.91	37.41	187.02^***^	41.20	84.25 ± 14.57b	122.71	HOH20

$\sigma_{G}^{2}$ denotes the genotypic variance, $\sigma_{e}^{2}$ the error variance, H^2^ the heritability within the treatments, $\;\sigma_{G\times T}^{2}$ the genotype-by-treatment interaction variance and r the correlation across treatments. The minimum values are abbreviated by ‘min’, the maximum values by ‘max’ and the mean values with the standard deviation by ‘mean ± sd’. Means with shared letters are not significantly different (at p < 0.05, Tukey-test) across treatment and environments. Asterisks display significance of variance components as ns, > 0.05; *, 0.01 < p ≤ 0.05; **, 0.001 < p ≤ 0.01; ***, p ≤ 0.001.

**Figure 1:**
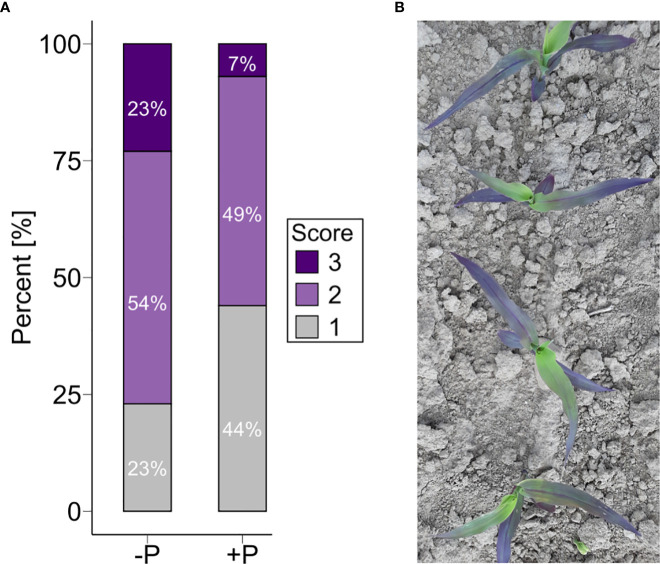
Proportion of plants with purple coloration of the leaves dependent on the P condition. **(A)** Purpleness evaluated in HOH20 by visual scoring on a plot basis from 1 = none to 3 = dark purple. P treatments are denoted as -P (without) and +P (with starter fertilization). **(B)** Discoloration of maize plants grown in -P conditions.

**Figure 2:**
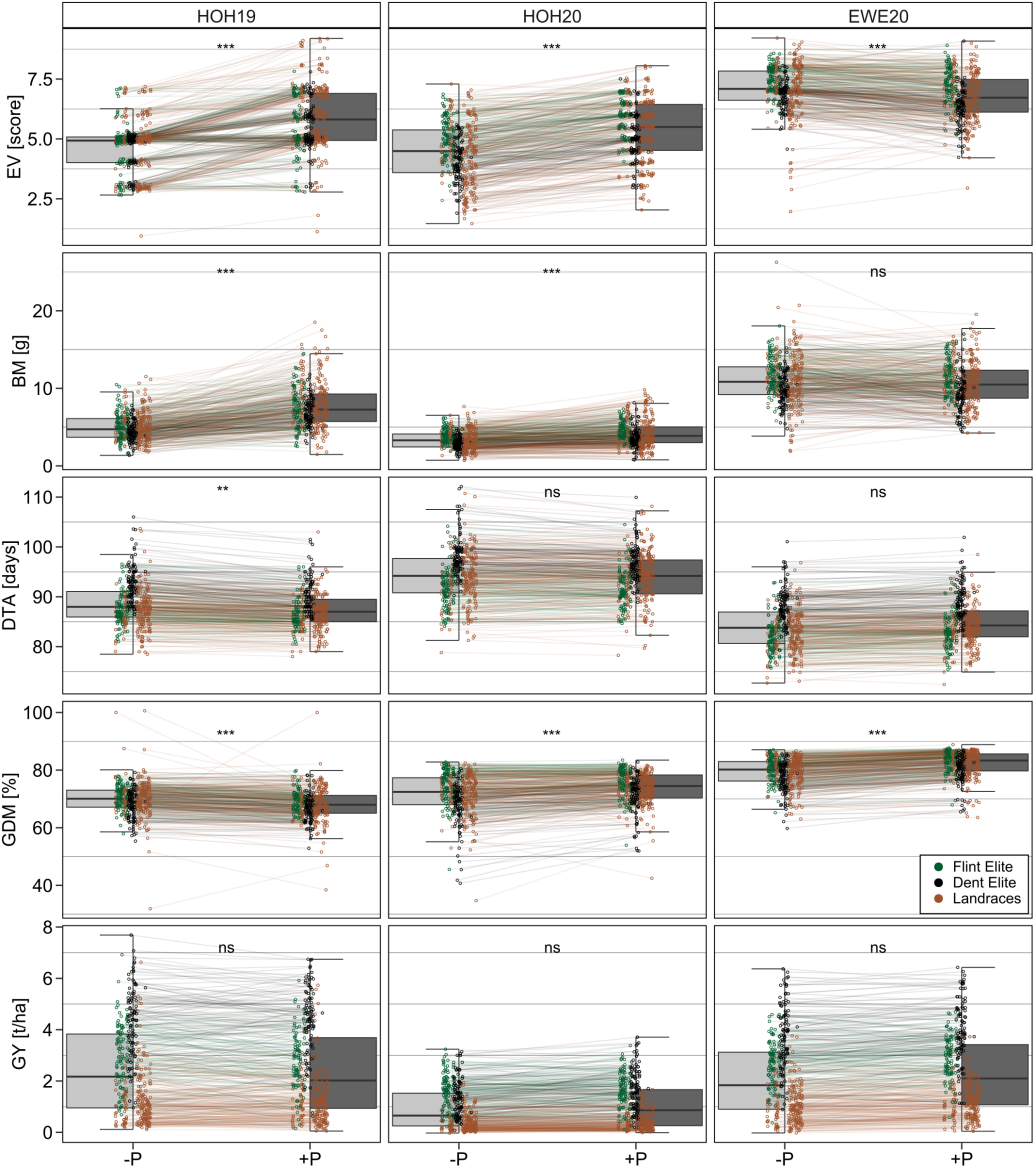
Trait differences between P treatments. Boxplots show trait distributions with (+P) or without (-P) starter fertilizer for each environment (HOH19, HOH20, EWE20). Note that genotypes in each treatment are connected by lines and colored as either Dent Elite, Flint Elite or landrace doubled haploid lines. Asterisks show significant differences between P treatment: ns, p > 0.05; *0.01 < p ≤ 0.05; **0.001 < p ≤ 0.01; ***p ≤ 0.001.

The differences in trait means revealed that early growth (PH1, PH3, PH4, EV) and biomass was significantly (p < 0.05) reduced in the -P treatment in HOH19 and HOH20 (**Table 2**). In addition, in HOH20, the treatment without starter fertilization resulted in 44 genotypes being moderately and 122 genotypes being severely purple discolored compared to their normal appearance in the fertilized +P treatment (**Figure 1**). In EWE20, by contrast, there were no such treatment differences in early traits. Traits measured in later plant development, including grain yield, were not affected by the treatment or showed differing responses depending on the environment (**Figure 2**, for other traits see **Figure S2**). For example, the time to male flowering (DTA) was significantly less under the +P condition than under -P condition in HOH19, even though this difference amounted to only 1.24 days, whereas no significant difference was observed in the other two environments. The percentage of grain dry matter content as a measure of maturity was lower under the +P treatment in HOH19 and also the lowest across all environments. In contrast, for HOH20 and EWE20, plants under -P treatment had lower grain dry matter content with a mean difference of 2.04 and 2.85%, respectively. For all environments, grain yield was not significantly influenced by P fertilization but was generally slightly higher in the +P treatment with an increase up to 0.17 t/ha in EWE20.

**Figure 3:**
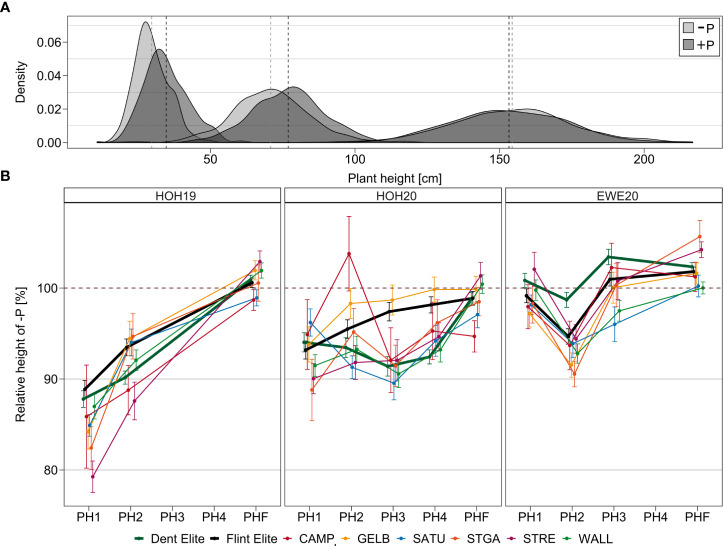
Response of plant height to P starter fertilizer. **(A)** Density plot of plant height distributions in HOH19 at three different time points (PH1, PH2, PHF) in control (-P) and starter fertilized (+P) conditions. Mean values are marked by a dashed line. **(B)** Relative plant height of genotypes under -P compared to +P treatment in HOH19, HOH20 and EWE20. Bars represent the standard deviation. The dashed line represents the same plant height in -P and +P treatment. Plant height was recorded at three (HOH19), four (EWE20) or five different time points (HOH20) (PH1, PH2, PH3, PH4, PHF). Populations are abbreviated as follows: Campan Galade (CAMP), Gelber Badischer Landmais (GELB), Satu Mare (SATU), St Galler Rheintaler (STGA), Strenzfelder (STRE) and Walliser (WALL).

The temporal dynamics of the difference between -P and +P during maize development are best illustrated with HOH19. When all plant height measurements from early development to final plant height (PH1, PH2, PH3, PH4, PHF) are considered, it becomes apparent that the difference between the two treatments is most pronounced at the early stage and becomes negligible with progressing plant development (**Figure 3**). The average relative plant height of genotypes under -P compared to +P treatment in HOH19 changed from 79.3% (PH1) to 102.9% (PHF). This effect of a progressively reduced difference was most pronounced for HOH19, less so for HOH20 and was almost absent for EWE20. In HOH20, Dent elite lines and the doubled haploid lines of the landrace “Gelber Badischer Landmais” followed this trend, whereas the relative height of other landrace doubled haploid lines and the Flint elite lines varied around 90% until PH4 or even until PHF. In EWE20, genotypes only showed a decreased performance under -P conditions for PH2, but were comparable in height at all other developmental stages.

**Figure 4:**
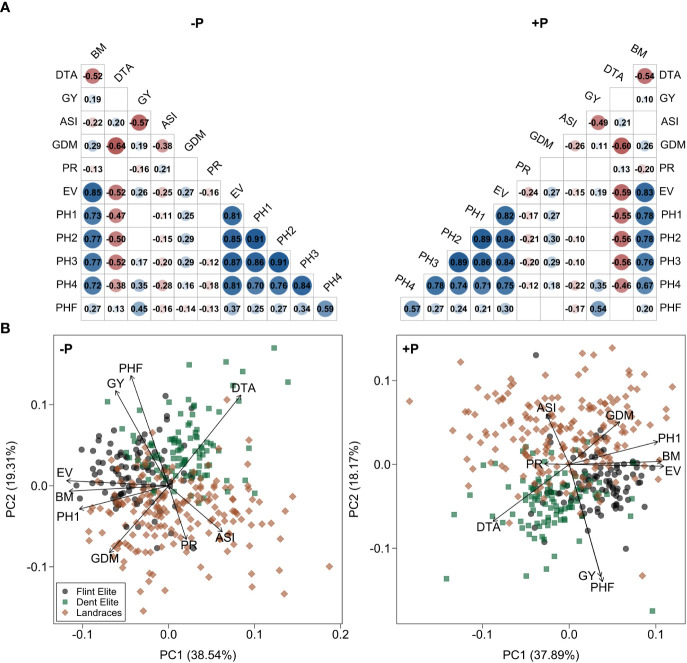
Relationships among traits in HOH20. **(A)** Correlation matrix and **(B)** principal component analysis of the lines based on phenotypic trait data. Results are shown without (-P) and with (+P) starter fertilizer. Blank fields in **(A)** are non-significant correlations. The genotypes in **(B)** are assigned to their material group as Dent Elite, Flint Elite and landrace doubled haploid lines.

There was a significant (p < 0.01) positive correlation between the two P treatments for all traits. The highest correlation was observed for grain yield (r = 0.96) and the lowest for biomass (r = 0.62) (**Table 2**). Correlations between traits measured in early stages, i.e. biomass, plant height and early vigor, were all highly positive, but these early stage traits showed little relation to grain yield. Grain yield displayed a negative correlation with anthesis-silking interval, a positive but rather weak association with days to anthesis and a positive correlation with final plant height. These associations between traits were almost identical between both treatments (**Figure 4A**, **S3A, S4A**).

### 3.2 Performance of diverse maize material in response to starter fertilizer

Regarding the relative performance of the elite Flint, Dent and landrace doubled haploid lines under the +P and -P conditions, general patterns could be observed in each environment, but also variations among them (**Tables S1–3**). For example, while the mean biomass of elite Dent and elite Flint lines decreased by 2.49 g and 2.23 g, respectively, without starter fertilizer in HOH19 (0.75 g and 0.72 g in HOH20), the difference between the mean biomass over all doubled haploid lines from landraces decreased more strongly by 3.47 g (0.94 g in HOH20). The same was true for plant height PH1 in HOH19, where the decrease without starter fertilizer was 6.52 cm for the landrace doubled haploid lines compared with 3.98 cm and 4.16 cm for the elite Dent and elite Flint lines, respectively. By contrast, grain yield and days to anthesis in HOH20 were most reduced in Dent lines compared to the other population groups.

To further characterize the contrasting phenotypic response of the various population groups, we performed a principal component analysis (**Figure 4B**, **S3B, S4B**). For all environments, the elite lines and landrace doubled haploid lines form partially overlapping but distinct clusters, for which the landrace doubled haploid lines cover a broader phenotypic space than the elite lines. The elite lines were characterized by higher values for grain yield, final plant height and particularly the Dent lines by late flowering. On the other hand, the landrace doubled haploid lines were distinguished by a long anthesis-silking interval, high grain dry matter content and, together with the elite Flint lines, a superior early performance. Although clustering between treatments was comparable, the change to the violet discoloration was noticeable, which had a more substantial contribution to the principal component under -P than +P conditions.

**Figure 5:**
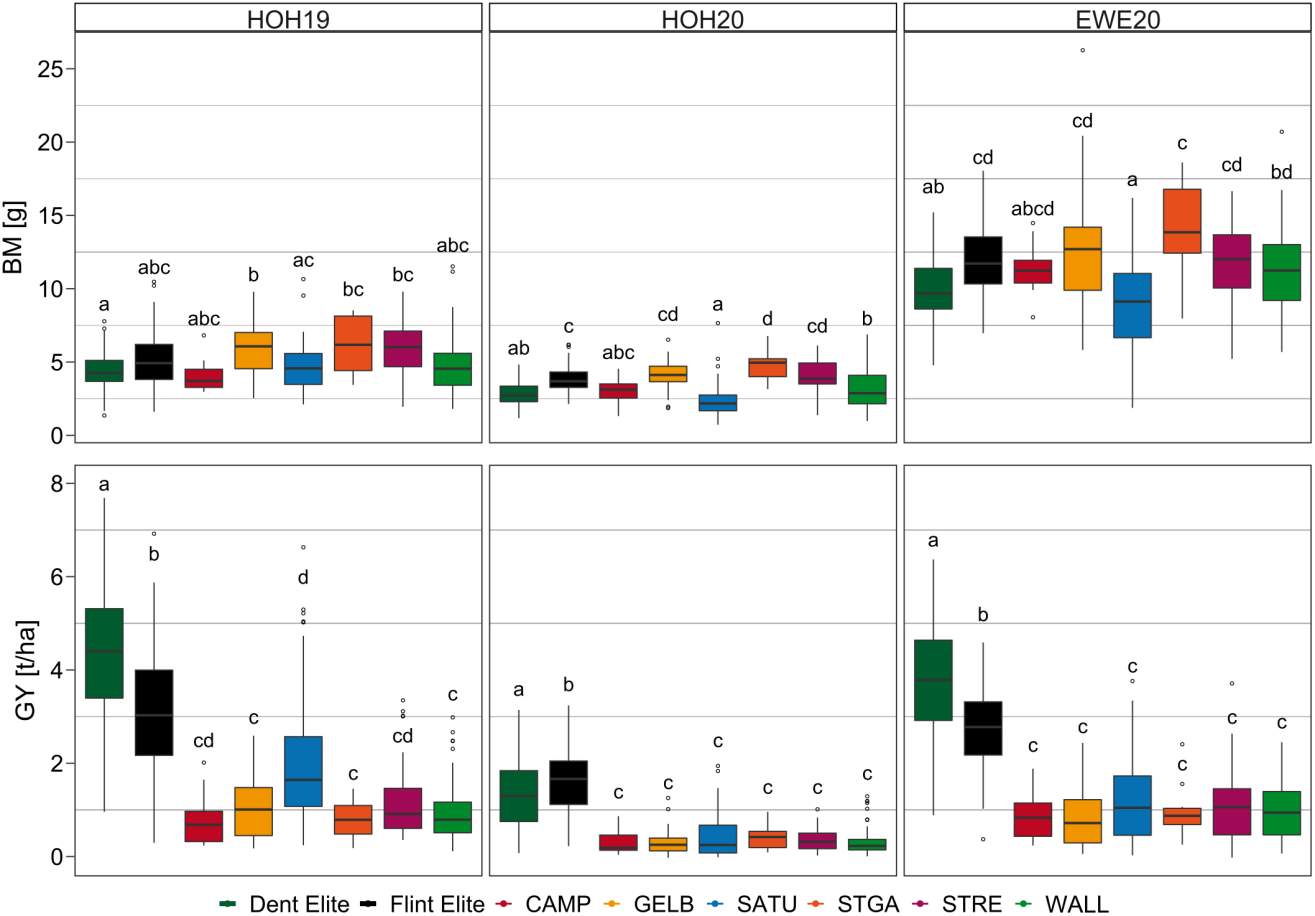
Biomass (BM) and grain yield (GY) values for single population groups by environment (HOH19, HOH20, EWE20) in -P. Population groups are divided into Dent and Flint elite lines and landrace doubled haploid lines. Note that landrace doubled haploid lines are further split up in their original six European Flint landraces. CAMP = Campan Galade, GELB = Gelber Badischer Landmais, SATU = Satu Mare, STGA = St Galler Rheintaler, STRE = Strenzfelder, WALL = Walliser. Means with shared letters were not significantly different using least significant difference.

The comparison between elite Flint, Dent and landrace doubled haploid lines under -P conditions, as a target scenario for a future agriculture, revealed significant differences in their agronomic performance for almost all traits within and across environments (**Tables S1–3**). Discrepancies between the population groups were particularly prominent for grain yield, as for example in HOH19 the mean value over all landrace doubled haploid lines was up to 3.19 t/ha lower than that of the elite Dent lines and 1.89 t/ha lower than the elite Flint lines. By contrast, the performance of Flint elite lines and landrace doubled haploid lines in early development, reflected in biomass and plant height values, was mostly superior to that of the Dent lines and, in some instances, the landrace doubled haploid lines also performed better than the Flint elite lines (**Figure 5**). The substantial variation in early plant development present in the landrace doubled haploid lines compared to the elite material is also illustrated by the larger genotypic variance component and phenotypic range. In contrast, the most extensive genotypic variation for grain yield was observed for the elite Flint and Dent material (**Tables S1–3**).

## 4 Discussion

The phenotypic response to reduced P fertilizer input was assessed in a multi-environment field trial for 399 maize genotypes representing elite Dent, elite Flint and doubled haploid European Flint landrace lines. This work aimed to evaluate the genetic potential for reducing P fertilization in maize towards breeding more P efficient varieties.

### 4.1 P starter fertilizer can affect early plant development

The response of the maize lines to the starter fertilizer treatment can be generalized insofar as it affected the early growth, but interestingly this varied with the environment. The performance of early growth-related traits (PH1, PH2, PH3, EV, PR, BM) was substantially improved under P fertilization in HOH19, had less effect in HOH20 and no effect in EWE20 (**Figures 2**, **3**). This illustrates the beneficial role that P starter fertilization can have even under optimal to high P saturation in the soil. The timepoint of P application is decisive for its impact on growth and yield since P stress is the most detrimental during the initial growth stages (Gericke, 1924; Römer and Schilling, 1986; Grant et al., 2001). Our results for plant height underline this clearly, as an advantage of the starter fertilization was only visible for early measurements (**Figure 3**). The nutrient requirement of maize is exceptionally high at the early developmental stages as there is a rapid daily increase in the dry biomass of the aboveground plant. In studies with maize at the ~BBCH V4 stage, growth under P deficiency was impaired by limited cell production and cell division rates (Assuero et al., 2004). Likewise, in wheat, P fertilization at 24 and 46 days of growth increased the relative growth rate considerably more than at a later application (Akhtar et al., 2011). The intra-plot variation for the plant height (PH1) measurements has shown that the growth in HOH was more homogenous within plots that received the starter fertilization. This variability among genetically identical plants could indicate small-scale variation of P availability in the soil, affecting growth on a single plant level, or alternatively reflects intra-plot heterogeneity with regard to other stress factors that becomes more pronounced when the plants lack the additional P from the starter fertilization.

An important question that arises from this is whether the possible negative influence of omitting the starter fertilizer on early growth stages has a subsequent impact on grain yield. We found that there was no significant difference between the two P treatments regarding grain yield, although for HOH20 and EWE20, the yield was slightly higher for +P (**Table 2**). We therefore conclude that the P content in the soil was high enough at the start of the growing period (**Table 1**) to support normal growth after the early development, a common observation in soils with high P availability (Roth et al., 2003; Subedi and Ma, 2009; Halilou et al., 2020). The legacy P reserves created by the liberate use of fertilizer in many Central European agriculturally used soils could provide nutrient uptake for a considerable time (Doydora et al., 2020). Additionally, studies have shown that P fertilization in combination with nitrogen was the most effective in increasing yield, while P on its own had little effect (Buttery et al., 1987; Osborne et al., 2004; Weiß et al., 2021). Since there was no significant increase of grain yield in our field trial, the higher financial expenses, particularly with the increased fertilizer prices, would not have been compensated for the farmers. The reduction in P application by omitting the starter fertilization could, therefore, not only benefit the environment but might also increase the return on investment for farmers.

What has been observed in later stages of development were differences in grain dry matter content, which can be taken as a proxy for physiological maturity. For HOH20 and EWE20, grain dry matter content was higher in plots treated with the starter fertilizer, which is in line with previous results (Shanka et al., 2015; Amanullah et al., 2016). We conclude that for these environments, the starter P application accelerated maturation, likely associated with its supporting role in diverse physiological processes (Ahemad et al., 2009). However, contrasting that, in HOH19, grain dry matter content was higher in plots without starter fertilizer, which has also been reported before. Zelalem et al. (2009) and Ojala et al. (1990) concluded that maturity might be delayed by excessive vegetative growth of the crops. Thus, the effect of P starter fertilization on maturity can also not be generalized but depends on the environmental conditions.

### 4.2 The effectiveness of P starter fertilization is dependent on the environment

Another critical question is why the effect of the starter fertilization not only varies in magnitude but is also only found in some environments. An answer to this can be well seen in the purple coloration of leaves observed in our study (**Figure 1**). This discoloration is due to an accumulation of anthocyanins, that in HOH19 was much more pronounced in the treatment without starter fertilizer. The build-up of anthocyanins under P stress decreases chlorophyll production, which obstructs photosynthesis (Henry et al., 2012). While the purple colorations can also have genetic reasons (Lafitte et al., 1997), as illustrated by the fact that some genotypes showed the same purple color in both treatments, many genotypes only showed the coloration in the P-reduced treatment. This observation suggests this phenomenon to be a consequence of P shortage. Still, as this anthocyanin accumulation under -P conditions was not observed in every environment, it is most likely attributable to the effect of cold temperature which was better tolerated by the plants given the starter fertilization (Cobbina and Miller, 1987). This illustrates that the starter fertilization not only prevents P stress but leads to a more vigorous growth during early stages, which in turn improves the abiotic stress tolerance of the plants, particularly the cold tolerance, which has been a major goal in European maize breeding for decades.

In conclusion, the effect of starter fertilization depends on the environmental conditions. Under stressful conditions, it can have a positive, protective effect, while under favorable conditions, growth is not impaired under -P and the starter fertilizer does not provide an advantage. While the omitted P fertilization had an evident effect in HOH19/20 on traits until the BBCH V6 stage, genotypes grown in EWE20 showed no apparent response to the treatment. This location has favorable climatic conditions for maize cultivation, being 1.3 degrees warmer during the growing season while also having the recommended P soil content (**Table 1**). These beneficial conditions resulted in higher values of the final plant height and a shorter anthesis-silking interval than in HOH (**Table 2**). As mentioned, the positive effects of P fertilization are more apparent under environmental stress by, for example, offsetting the impact of cold temperature (Gates et al., 1973). Thus, the combination of favorable environmental conditions and high P content in the soil, even without starter fertilizer, is likely why plants under the control treatment showed no disadvantage in growth in EWE. An alternative explanation may be that P uptake in EWE20 was hindered and consequently, no treatment differences could be observed. Arguments for this would be the comparably low pH value in EWE20 of 5.6, potentially leading to rapid P fixation by aluminum, while the optimal pH for P uptake lies at around 6.5 (Penn and Camberato, 2019). On top of that, P uptake by plants is generally hindered by aridity. The already slow diffusion of P ions under normal conditions occurs over water-filled pores or soil particle-wetting water films (Mahtab et al., 1971), but EWE20 was the driest location at the beginning of the growing season, possibly limiting P availability (**Table 1**). Taken together, these results show how strongly the environment, i.e. water availability, nutrients, soil conditions and abiotic stress around the plant, influences the effectiveness of P fertilization.

### 4.3 Can we breed varieties that allow to abandon the application of starter fertilizer?

An important question that arises from these findings is if there is genotypic variation for the response to the starter fertilizer. We have seen that starter fertilization can buffer adverse environmental conditions by improving the plants’ stress response. Thus, its application serves as an insurance for the farmer as the exact climatic conditions for the coming season cannot be predicted. However, if varieties could be bred that perform well without the starter fertilization even under adverse environmental conditions, the application of starter fertilizer could be abandoned.

A strong positive correlation between the treatments was found for all traits in all environments (**Table 2**) as well as comparable associations between traits (**Figure 4A**, **Figures S3A, S4A**). This indicates a similar genetic control underlying trait expression under the two P conditions, consistent with the observed small genotype-by-treatment interaction. A highly important trait is grain yield and the positive association between the treatments means that selection under starter fertilized conditions also identifies those genotypes that achieve high yields under conditions without the starter fertilizer. Thus, breeding has so far already worked in the right direction. The question is whether there is a favorable P condition for breeding P efficient genotypes. The heritability and the genotypic variance were very similar under both conditions, which is in contrast to Blum (1988) and Atlin and Frey (1990), who observed that non-stressed conditions allowed for a greater genotypic variation. Our results suggest that there appears to be no advantage of performing selection for grain yield under one or the other P condition. Nevertheless, if future agriculture will be based on a reduced fertilizer use, selection is preferably performed under the target conditions. Moreover, despite the low genotype-by-treatment interaction, breeding should exploit the extremes, which in this case means genotypes that perform well and stable across P conditions. Our results illustrate that this is possible as there are lines that show a comparably slight reduction and a generally good agronomic performance when grown without starter fertilizer, even under adverse conditions as experienced in HOH19. The next step should be to evaluate genotypic variation in the uptake and allocation of P in the plant as well as the storage of phytic acid in the grain, as this impacts nutritional aspects and is utilized during germination and seedling growth (Oatway et al., 2001).

### 4.4 Potential of elite and doubled haploid landrace lines for reduced fertilizer application

Selection in maize breeding was so far made under well-supplied soils, which generally improved the yield potential as evidenced by the fact that the elite lines exceeded all doubled haploid landrace lines in grain yield under both P conditions. We observed significantly lower early trait values for elite Dent lines, presumably due to their lower cold tolerance (**Figure 5**) (Unterseer et al., 2016). The late maturity of Dent lines, also observed in our study, could allow for a more extended time period to accumulate P before flowering and grain formation (**Tables S1–3**) (Lafitte et al., 1997). However, it can also be critical due to the lower grain dry matter content at harvest time.

While high grain yield characterizes elite material, landraces can show notable yield stability in stressed environments (Ficiciyan et al., 2018). Moreover, it could be hypothesized that valuable genetic variation for P efficiency exists in landraces as these predate the intensive application of P fertilizer in agriculture. However, considering the observed smaller genotype-by-treatment interaction of the elite lines than of the landrace doubled haploid lines, this illustrates a stronger and less predictable response of the latter to the starter fertilizer at least for some traits (**Tables S1–3**). Thus, our results do not support the hypothesis that landrace doubled haploid lines are generally more stable in their performance under different P conditions than elite lines. This may be because landraces are only adapted to a specific target environment, while elite lines undergo intensive breeding efforts and are routinely tested in multi-environment trials. Therefore, only lines that show a stable performance across various environments are selected, which may have generally reduced their genotype-by-environment interaction (Gage et al., 2017).

Nevertheless, the landrace doubled haploid lines may harbor genetic variation not present in elite breeding material that could be valuable for breeding for lower P conditions. As we have observed, the most critical phase is early development. Improving this will also enhance the abiotic stress tolerance under reduced P levels. Regarding those early development traits, some landrace doubled haploid lines performed as well or even better than the elite groups (**Figure 5**). The favorable seedling performance is likely due to their considerable local adaptation, faster development and cold tolerance (Peter et al., 2009).

The use of just a few founder lines as a basis for the European elite Flint pool is the reason for the comparably low genetic diversity, while the Dent pool has been expanded by material from the US Cornbelt and Southern Europe (Böhm et al., 2017). Thus, individual landrace doubled haploid lines may hold potential for maize breeding and introgression of traits into the Flint elite pool, though this must be well considered given their often lower agronomic fitness and yield as well as the quantitative nature of the target traits. In addition, landrace doubled haploid lines may hold greater potential for more severe P stress that we may encounter in the future, which warrants further research. Furthermore, knowledge about quantitative trait loci for direct or indirect P efficiency traits will facilitate a molecular comparison with elite material as well as a more targeted and thus faster introgression of favorable characteristics.

## 5 Conclusions

Our study aimed to dissect the phenotypic response of diverse maize breeding material to the application of P starter fertilizer under temperate European conditions. Our results demonstrate that under typical Central European soil conditions, a reduction of P fertilization by omitting the starter fertilizer can affect early traits under adverse environmental conditions, while late development remained largely unaffected. The standard application of starter fertilizer seems to be more of an insurance against abiotic stress during the early stages than for increasing yield. However, we observed substantial genotypic variation for this response to the two P levels, which lays the foundation for breeding of varieties that show good performance under varying P conditions. The landrace doubled haploid lines generally responded more strongly to differences in P fertilization and their introgression in elite material must therefore be carefully considered. Nevertheless, due to their adaptation, landraces may harbor beneficial properties with regard to early development. Notably, this work is based on maize lines and further research is required to assess how this response to P starter fertilization is expressed in hybrids. In conclusion, breeding for P efficiency will gain further importance in future when the legacy P content in the soil is lowered due to restricted fertilizer use and efforts to improve this trait should consequently start today.

## Data availability statement

The raw data supporting the conclusions of this article will be made available by the authors, without undue reservation.

## Author contributions

SR: Formal Analysis, Investigation, Data Curation, Writing – Original Draft Preparation, Writing – Review and Editing, Visualization; TW: Conceptualization, Methodology, Writing – Original Draft Preparation, Writing – Review and Editing, Visualization, Supervision, Funding Acquisition; TMW: Data Curation, Writing – Review and Editing; DL, WXL, WS, AM, VH, and WLL: Writing – Review and Editing. All authors contributed to the article and approved the submitted version.
