# Supplementary material for: Can we abandon phosphorus starter fertilizer in maize? Results from a diverse panel of elite and doubled haploid landrace lines of maize (Zea mays L.)
Source: Front Plant Sci. 2022 Dec 16;13:1005931. doi: 10.3389/fpls.2022.1005931 (PMC9800985; doi:10.3389/fpls.2022.1005931)
Supplement: Supplementary file 1 [file DataSheet_1.docx]

Supplementary Material

# **Supplementary Tables**

**Table S1** Population-specific summary statistics for the evaluated phenotypic traits (denoted by "-" for -P and "+" for +P treatment) measured in HOH19. $\sigma_{G}^{2}$ denotes the genotypic variance, $H^{2}$ the heritability and $\sigma_{G\times T}^{2}$ the genotype-by-treatment interaction variance. The minimum values are abbreviated by 'min', the maximum values by 'max' and the mean values with the standard deviation by 'mean **±** sd'. Means with shared letters are not significantly different (at p < 0.05) in performance across treatments. Asterisks display significance of variance components as ns, > 0.05; *, 0.01 < p ≤ 0.05; **, 0.001 < p ≤ 0.01; ***, p ≤ 0.001. Populations are abbreviated as follows: Elite Dent (ED), Elite Flint (EF), Campan Galade (CG), Gelber Badischer Landmais (GB), Satu Mare (SM), St Galler Rheintaler (RT), Strenzfelder (SF) and Walliser (WA).

|  |  | - BM1 | + BM1 | - DTA | + DTA | - EV | + EV | - Gyield | + Gyield | - PH1 | + PH1 | - ASI | + ASI | - GDM | + GDM | - PH2 | + PH2 | - PHF | + PHF |
| --- | --- | --- | --- | --- | --- | --- | --- | --- | --- | --- | --- | --- | --- | --- | --- | --- | --- | --- | --- |
| ED  (n = 100) | max | 7.78 | 11.80 | 106.00 | 101.50 | 5.28 | 7.80 | 7.69 | 6.74 | 35.64 | 42.32 | 6.50 | 6.57 | 79.18 | 78.25 | 82.76 | 96.06 | 202.80 | 216.83 |
|  | mean | 4.42 ± 1.24a | 6.91 ± 1.75b | 92.65 ± 3.85a | 90.33 ± 3.45b | 4.53 ± 0.74a | 5.53 ± 1.00b | 4.34 ± 1.41a | 4.37 ± 1.32a | 27.16 ± 3.69a | 31.14 ± 4.49b | 1.51 ± 1.87a | 1.89 ± 1.59a | 68.17 ± 5.01a | 66.57 ± 4.77b | 65.57 ± 8.76a | 72.82 ± 8.82b | 163.69 ± 17.63a | 162.64 ± 18.49a |
|  | min | 1.36 | 2.88 | 86.44 | 85.50 | 2.87 | 2.97 | 0.96 | 0.62 | 16.17 | 20.13 | -3.44 | -1.93 | 55.36 | 52.87 | 39.39 | 49.06 | 114.57 | 124.29 |
|  | *σ****^2^_G_*** | 0.44* | 1.40*** | 14.30*** | 11.50*** | 0.35*** | 0.69*** | 1.80*** | 1.63*** | 8.68*** | 14.77*** | 2.62*** | 1.72*** | 17.78*** | 12.84*** | 61.15*** | 60.87*** | 272.22*** | 306.84*** |
|  | *H****^2^*** | 0.33 | 0.53 | 0.97 | 0.97 | 0.67 | 0.73 | 0.94 | 0.95 | 0.68 | 0.77 | 0.76 | 0.70 | 0.71 | 0.65 | 0.83 | 0.81 | 0.89 | 0.92 |
|  | *σ****^2^_G×T_*** | < 0.01^ns^ | | < 0.01*** | | < 0.01^ns^ | | 0.06*** | | < 0.01^ns^ | | < 0.01^ns^ | | < 0.01^ns^ | | < 0.01^ns^ | | 5.47^ns^ | |
| EF (n = 100) | max | 10.48 | 14.47 | 96.54 | 96.00 | 7.12 | 7.82 | 6.92 | 5.92 | 46.39 | 47.44 | 8.51 | 8.10 | 77.94 | 79.86 | 105.97 | 103.87 | 198.82 | 200.81 |
|  | mean | 5.11 ± 1.71a | 7.34 ± 2.49b | 87.46 ± 3.20a | 86.56 ± 3.10b | 4.66 ± 1.01a | 5.51 ± 1.20b | 3.04 ± 1.26a | 2.80 ± 1.14a | 30.42 ± 4.95a | 34.58 ± 6.32b | 2.29 ± 1.83a | 2.60 ± 1.74a | 70.90 ± 3.82a | 69.48 ± 3.85b | 74.21 ± 11.31a | 79.80 ± 11.84b | 161.94 ± 14.95a | 161.20 ± 14.66a |
|  | min | 1.61 | 2.51 | 81.01 | 80.50 | 2.66 | 2.78 | 0.30 | 0.15 | 21.16 | 19.52 | -2.02 | -1.50 | 57.91 | 61.69 | 45.16 | 44.31 | 121.47 | 129.42 |
|  | *σ****^2^_G_*** | 1.54*** | 4.13*** | 9.73*** | 9.17*** | 0.79*** | 1.13*** | 1.43*** | 1.21*** | 19.10*** | 34.62*** | 2.45*** | 2.24*** | 7.22*** | 5.94*** | 110.51*** | 110.73*** | 182.73*** | 183.18*** |
|  | *H****^2^*** | 0.63 | 0.77 | 0.95 | 0.96 | 0.82 | 0.82 | 0.92 | 0.94 | 0.82 | 0.89 | 0.75 | 0.75 | 0.50 | 0.46 | 0.90 | 0.89 | 0.85 | 0.87 |
|  | *σ****^2^_G×T_*** | < 0.01^ns^ | | < 0.01^ns^ | | < 0.01^ns^ | | < 0.01*** | | < 0.01^ns^ | | < 0.01^ns^ | | < 0.01^ns^ | | 7.31** | | < 0.01^ns^ | |
| CAMP (n = 11) | max | 6.82 | 10.72 | 92.60 | 90.50 | 5.88 | 7.32 | 2.01 | 1.89 | 36.12 | 45.33 | 5.54 | 5.45 | 100.01 | 77.77 | 79.38 | 94.92 | 173.48 | 167.67 |
|  | mean | 4.13 ± 1.29a | 7.75 ± 2.09b | 84.72 ± 4.04a | 83.82 ± 3.68a | 4.03 ± 1.16a | 5.59 ± 1.45b | 0.81 ± 0.60a | 0.78 ± 0.60a | 27.75 ± 4.11a | 34.47 ± 7.38b | 2.81 ± 1.71a | 3.17 ± 1.78a | 73.97 ± 9.71a | 68.67 ± 5.97a | 63.86 ± 10.78a | 75.32 ± 13.09a | 144.14 ± 16.67a | 145.81 ± 13a |
|  | min | 2.97 | 5.39 | 79.04 | 78.00 | 2.80 | 3.00 | 0.23 | 0.18 | 23.14 | 21.94 | -0.50 | -0.09 | 65.20 | 62.06 | 49.75 | 60.06 | 119.25 | 121.60 |
|  | *σ****^2^_G_*** | 0.53^ns^ | 2.45^ns^ | 15.82*** | 13.20*** | 1.08*** | 1.67*** | 0.23** | 0.24*** | 13.22*** | 46.37*** | 2.03*** | 2.23*** | 85.15*** | 19.39* | 105.31*** | 152.69*** | 246.75*** | 139.63*** |
|  | *H****^2^*** | 0.37 | 0.66 | 0.97 | 0.97 | 0.86 | 0.87 | 0.66 | 0.75 | 0.76 | 0.91 | 0.71 | 0.76 | 0.92 | 0.73 | 0.90 | 0.92 | 0.89 | 0.84 |
|  | *σ****^2^_G×T_*** | < 0.01^ns^ | | < 0.01^ns^ | | < 0.01^ns^ | | < 0.01^ns^ | | < 0.01^ns^ | | < 0.01^ns^ | | 44.53*** | | 1.93^ns^ | | < 0.01^ns^ | |
| GELB (n = 32) | max | 9.80 | 15.66 | 96.05 | 92.00 | 7.15 | 9.11 | 2.59 | 3.11 | 48.95 | 60.62 | 8.13 | 9.87 | 87.46 | 77.32 | 107.42 | 115.28 | 211.97 | 186.72 |
|  | mean | 6.01 ± 1.95a | 10.35 ± 2.96b | 86.78 ± 3.00a | 85.89 ± 3.03a | 5.50 ± 1.14a | 6.98 ± 1.40b | 1.05 ± 0.68a | 1.34 ± 0.73a | 36.45 ± 5.51a | 43.82 ± 7.54b | 3.87 ± 2.39a | 3.85 ± 2.48a | 69.99 ± 4.65a | 67.94 ± 3.42a | 80.98 ± 13.28a | 87.13 ± 14.02a | 158.79 ± 21.39a | 155.70 ± 18.24a |
|  | min | 2.54 | 4.94 | 81.52 | 79.00 | 2.98 | 2.81 | 0.18 | 0.25 | 23.62 | 24.81 | -0.02 | 0.35 | 63.01 | 60.79 | 37.88 | 53.66 | 114.45 | 116.99 |
|  | *σ****^2^_G_*** | 2.02*** | 6.75*** | 8.44*** | 8.78*** | 1.03*** | 1.65*** | 0.32*** | 0.45*** | 23.23*** | 52.00*** | 4.85*** | 5.37*** | 4.70* | 3.12^ns^ | 145.26*** | 182.80*** | 419.04*** | 302.22*** |
|  | *H****^2^*** | 0.69 | 0.84 | 0.94 | 0.96 | 0.86 | 0.87 | 0.73 | 0.85 | 0.85 | 0.92 | 0.85 | 0.88 | 0.39 | 0.31 | 0.92 | 0.93 | 0.93 | 0.92 |
|  | *σ****^2^_G×T_*** | 2.42*** | | < 0.01*** | | < 0.01*** | | < 0.01^ns^ | | < 0.01*** | | 0.77** | | < 0.01^ns^ | | 11.10* | | 13.72^ns^ | |
| SATU (n = 53) | max | 10.66 | 18.53 | 103.67 | 103.00 | 6.93 | 7.19 | 6.63 | 5.73 | 38.63 | 48.69 | 11.47 | 14.30 | 78.84 | 100.00 | 98.22 | 115.41 | 206.05 | 197.93 |
|  | mean | 4.69 ± 1.75a | 6.83 ± 3.20b | 88.08 ± 4.66a | 87.53 ± 4.07a | 4.09 ± 1.16a | 5.02 ± 1.31b | 2.05 ± 1.49a | 1.68 ± 1.43a | 24.88 ± 4.94a | 28.67 ± 6.19b | 3.73 ± 2.86a | 4.60 ± 3.38a | 68.95 ± 4.21a | 67.20 ± 6.52a | 64.73 ± 13.72a | 66.90 ± 14.79a | 143.43 ± 25.71a | 145.03 ± 24.18a |
|  | min | 2.11 | 1.47 | 78.98 | 79.00 | 0.95 | 1.13 | 0.24 | 0.06 | 16.82 | 10.79 | -3.11 | -1.00 | 59.73 | 57.65 | 19.85 | 21.12 | 93.08 | 86.18 |
|  | *σ****^2^_G_*** | 2.86*** | 8.20*** | 21.12*** | 16.17*** | 1.08*** | 1.34*** | 2.07*** | 1.97*** | 17.87*** | 32.55*** | 7.36*** | 10.48*** | 10.49*** | 35.62*** | 118.09*** | 164.29*** | 621.16*** | 514.31*** |
|  | *H****^2^*** | 0.76 | 0.87 | 0.98 | 0.98 | 0.86 | 0.84 | 0.95 | 0.96 | 0.81 | 0.88 | 0.90 | 0.93 | 0.59 | 0.84 | 0.91 | 0.92 | 0.95 | 0.95 |
|  | *σ****^2^_G×T_*** | 0.24*** | | < 0.01*** | | < 0.01^ns^ | | < 0.01*** | | < 0.01^ns^ | | 1.35*** | | 6.34** | | 5.92^ns^ | | 8.84^ns^ | |
| STGA (n = 14) | max | 8.53 | 14.71 | 89.02 | 87.50 | 7.07 | 9.20 | 1.45 | 1.72 | 43.26 | 53.42 | 5.48 | 8.75 | 77.85 | 76.63 | 104.59 | 95.80 | 148.45 | 141.78 |
|  | mean | 6.14 ± 1.89a | 10.71 ± 2.95b | 82.88 ± 2.62a | 82.21 ± 2.19a | 5.22 ± 1.16a | 6.91 ± 1.49b | 0.80 ± 0.41a | 0.78 ± 0.45a | 34.31 ± 5.58a | 41.91 ± 7.35b | 2.81 ± 1.52a | 3.49 ± 1.79a | 72.79 ± 2.96a | 71.23 ± 2.92a | 80.91 ± 13.58a | 83.91 ± 9.95a | 125.95 ± 11.46a | 125.42 ± 9.24a |
|  | min | 3.44 | 5.28 | 79.96 | 79.50 | 2.93 | 4.17 | 0.18 | 0.23 | 25.90 | 29.05 | 0.02 | 1.45 | 65.67 | 67.63 | 59.74 | 64.45 | 108.13 | 110.52 |
|  | *σ****^2^_G_*** | 2.47*** | 6.07*** | 6.41*** | 4.46*** | 1.22*** | 1.90*** | 0.02^ns^ | 0.12* | 27.15*** | 48.41*** | 1.42** | 2.50*** | < 0.01^ns^ | < 0.01^ns^ | 150.17*** | 84.56*** | 91.33*** | 56.21*** |
|  | *H****^2^*** | 0.73 | 0.83 | 0.93 | 0.92 | 0.87 | 0.88 | 0.16 | 0.59 | 0.87 | 0.92 | 0.63 | 0.77 | 0.00 | 0.00 | 0.92 | 0.86 | 0.74 | 0.68 |
|  | *σ****^2^_G×T_*** | 3.42*** | | < 0.01^ns^ | | < 0.01^ns^ | | < 0.01^ns^ | | < 0.01* | | 0.80* | | < 0.01^ns^ | | 20.25* | | 0.81^ns^ | |
| STRE (n = 30) | max | 9.80 | 17.51 | 99.04 | 93.88 | 7.18 | 9.07 | 3.35 | 2.49 | 50.25 | 60.34 | 7.94 | 8.97 | 100.59 | 82.20 | 108.99 | 119.61 | 170.20 | 170.01 |
|  | mean | 5.88 ± 2.10a | 9.15 ± 3.40b | 87.93 ± 3.65a | 85.91 ± 2.79b | 4.95 ± 1.16a | 6.19 ± 1.46b | 1.24 ± 0.90a | 1.13 ± 0.63a | 31.65 ± 6.94a | 39.90 ± 8.34b | 2.18 ± 2.05a | 2.76 ± 2.10a | 73.52 ± 7.13a | 71.83 ± 5.67a | 72.68 ± 16.33a | 81.97 ± 14.47b | 148.16 ± 11.18a | 144.30 ± 11.75a |
|  | min | 1.95 | 2.34 | 78.89 | 80.50 | 2.92 | 3.01 | 0.35 | 0.19 | 20.01 | 22.28 | -1.95 | -1.06 | 60.24 | 51.58 | 42.09 | 54.67 | 123.01 | 123.86 |
|  | *σ****^2^_G_*** | 2.78*** | 9.40*** | 12.80*** | 7.16*** | 1.13*** | 1.76*** | 0.66*** | 0.30*** | 41.60*** | 63.57*** | 3.34*** | 3.74*** | 10.22*** | 17.02*** | 214.37*** | 158.71*** | 87.55*** | 108.80*** |
|  | *H****^2^*** | 0.76 | 0.88 | 0.96 | 0.95 | 0.87 | 0.88 | 0.85 | 0.79 | 0.91 | 0.94 | 0.80 | 0.84 | 0.59 | 0.71 | 0.95 | 0.92 | 0.73 | 0.80 |
|  | *σ****^2^_G×T_*** | 2.44*** | | < 0.01*** | | < 0.01^ns^ | | < 0.01* | | < 0.01*** | | 0.77** | | < 0.01^ns^ | | 22.45*** | | 6.77^ns^ | |
| WALL (n = 59) | max | 11.52 | 16.69 | 97.12 | 95.50 | 7.06 | 9.18 | 2.98 | 2.41 | 45.81 | 52.41 | 13.52 | 12.06 | 77.21 | 75.45 | 99.67 | 103.32 | 179.41 | 171.76 |
|  | mean | 4.85 ± 2.11a | 7.70 ± 2.89b | 87.52 ± 3.33a | 86.96 ± 2.98a | 4.75 ± 1.11a | 6.11 ± 1.20b | 0.96 ± 0.66a | 0.95 ± 0.61a | 32.13 ± 5.48a | 37.51 ± 6.85b | 5.47 ± 3.19a | 5.76 ± 3.19a | 68.45 ± 7.15a | 66.53 ± 7.30a | 72.07 ± 11.14a | 78.41 ± 10.88b | 145.20 ± 14.29a | 142.76 ± 14.23a |
|  | min | 1.81 | 1.79 | 78.48 | 80.00 | 2.83 | 3.99 | 0.11 | 0.05 | 22.95 | 23.94 | 0.59 | 0.46 | 31.88 | 38.43 | 46.07 | 45.74 | 116.46 | 115.11 |
|  | σ**^2^_G_** | 3.28*** | 5.56*** | 10.55*** | 8.45*** | 0.98*** | 1.09*** | 0.31*** | 0.26*** | 25.03*** | 42.20*** | 9.15*** | 9.30*** | 43.89*** | 36.06*** | 95.51*** | 99.52*** | 167.05*** | 173.42*** |
|  | H**^2^** | 0.79 | 0.82 | 0.96 | 0.95 | 0.85 | 0.81 | 0.73 | 0.77 | 0.86 | 0.91 | 0.92 | 0.93 | 0.86 | 0.84 | 0.89 | 0.88 | 0.84 | 0.87 |
|  | σ**^2^_G×T_** | < 0.01** | | < 0.01*** | | < 0.01* | | < 0.01^ns^ | | < 0.01** | | 0.27^ns^ | | < 0.01^ns^ | | 7.10* | | 7.22^ns^ | |

**Table S2** Population-specific summary statistics for the evaluated phenotypic traits (denoted by "-" for -P and "+" for +P treatment) measured in HOH20. $\sigma_{G}^{2}$ denotes the genotypic variance, $H^{2}$ the heritability and $\sigma_{G\times T}^{2}$ the genotype-by-treatment interaction variance. The minimum values are abbreviated by 'min'. the maximum values by 'max' and the mean values with the standard deviation by 'mean **±** sd'. Means with shared letters are not significantly different (at p < 0.05. Tukey-test) in performance across treatments. Asterisks display significance of variance components as ns, > 0.05; *, 0.01 < p ≤ 0.05; **, 0.001 < p ≤ 0.01; ***, p ≤ 0.001. Populations are abbreviated as follows: Elite Dent (ED). Elite Flint (EF). Campan Galade (CG). Gelber Badischer Landmais (GB). Satu Mare (SM). St Galler Rheintaler (RT). Strenzfelder (SF) and Walliser (WA).

|  |  | - BM1 | + BM1 | - DTA | + DTA | - EV | + EV | - Gyield | + Gyield | - PH1 | + PH1 | - PR | + PR | - ASI | + ASI | - GDM | + GDM | - PH2 | + PH2 | - PH3 | + PH3 | - PH4 | + PH4 | - PHF | + PHF |
| --- | --- | --- | --- | --- | --- | --- | --- | --- | --- | --- | --- | --- | --- | --- | --- | --- | --- | --- | --- | --- | --- | --- | --- | --- | --- |
| ED  (n = 100) | max | 4.83 | 8.14 | 112.12 | 109.94 | 6.12 | 7.00 | 3.14 | 3.71 | 26.17 | 31.64 | 3.00 | 3.02 | 12.12 | 11.50 | 80.52 | 82.79 | 32.46 | 37.50 | 45.48 | 49.34 | 109.55 | 109.79 | 164.83 | 164.44 |
|  | mean | 2.83 ± 0.79a | 3.58 ± 1.23b | 98.43 ± 4.03a | 97.03 ± 3.65b | 4.09 ± 0.85a | 4.97 ± 0.89b | 1.34 ± 0.71a | 1.68 ± 0.81b | 20.09 ± 2.65a | 21.53 ± 3.20b | 1.87 ± 0.68a | 1.68 ± 0.63b | 4.59 ± 2.15a | 3.60 ± 2.00b | 67.90 ± 7.62a | 71.11 ± 6.27b | 23.78 ± 3.67a | 25.58 ± 3.77b | 31.04 ± 5.24a | 34.05 ± 4.89b | 76.70 ± 10.97a | 83.13 ± 10.74b | 128.85 ± 15.15a | 130.62 ± 14.21a |
|  | min | 1.17 | 0.77 | 90.26 | 88.89 | 1.90 | 2.95 | 0.07 | 0.18 | 13.60 | 14.60 | 1.00 | 0.97 | 0.03 | -0.50 | 40.73 | 51.94 | 14.21 | 17.21 | 18.59 | 21.95 | 52.06 | 54.85 | 89.07 | 102.17 |
|  | *σ****^2^_G_*** | 0.35*** | 0.89*** | 15.43*** | 12.50*** | 0.54*** | 0.62*** | 0.45*** | 0.61*** | 4.74*** | 6.46*** | 0.40*** | 0.33*** | 3.24*** | 3.05*** | 52.31*** | 36.42*** | 10.39*** | 9.87*** | 19.26*** | 17.30*** | 90.38*** | 86.97*** | 185.34*** | 162.26*** |
|  | *H****^2^*** | 0.62 | 0.64 | 0.97 | 0.95 | 0.81 | 0.81 | 0.91 | 0.94 | 0.70 | 0.67 | 0.88 | 0.84 | 0.72 | 0.76 | 0.92 | 0.94 | 0.82 | 0.73 | 0.76 | 0.76 | 0.81 | 0.80 | 0.87 | 0.83 |
|  | *σ****^2^_G×T_*** | 0.13* | | 0.92*** | | < 0.01^ns^ | | 0.08*** | | < 0.01^ns^ | | < 0.01** | | < 0.01^ns^ | | 1.56* | | 0.36^ns^ | | < 0.01^ns^ | | < 0.01^ns^ | | 3.51^ns^ | |
| EF (n = 100) | max | 6.18 | 7.32 | 104.16 | 104.48 | 6.87 | 7.50 | 3.24 | 3.38 | 32.87 | 34.39 | 3.00 | 3.00 | 9.17 | 6.03 | 82.81 | 83.48 | 37.58 | 40.14 | 51.03 | 49.00 | 115.28 | 115.86 | 161.58 | 166.35 |
|  | mean | 3.86 ± 0.86a | 4.58 ± 1.08b | 91.63 ± 4.04a | 91.63 ± 3.65a | 5.12 ± 0.82a | 5.96 ± 0.80b | 1.63 ± 0.69a | 1.69 ± 0.67a | 23.80 ± 3.10a | 25.72 ± 3.57b | 1.99 ± 0.57a | 1.61 ± 0.50b | 2.99 ± 1.93a | 2.57 ± 1.53a | 75.03 ± 6.12a | 76.46 ± 5.00a | 28.90 ± 3.87a | 30.41 ± 3.79b | 38.81 ± 5.15a | 39.97 ± 4.61a | 91.37 ± 10.44a | 93.43 ± 10.38a | 128.97 ± 14.04a | 130.82 ± 14.28a |
|  | min | 2.14 | 2.33 | 84.05 | 84.64 | 3.04 | 3.92 | 0.22 | 0.11 | 17.59 | 18.65 | 1.00 | 0.96 | 0.10 | 0.00 | 45.52 | 53.91 | 21.27 | 21.81 | 26.35 | 29.83 | 64.84 | 65.61 | 86.85 | 75.63 |
|  | *σ****^2^_G_*** | 0.45*** | 0.58*** | 15.59*** | 12.48*** | 0.51*** | 0.48*** | 0.43*** | 0.39*** | 7.20*** | 9.16*** | 0.27*** | 0.18*** | 2.40*** | 1.31*** | 23.02*** | 20.19*** | 12.24*** | 10.24*** | 19.68*** | 14.82*** | 80.65*** | 80.86*** | 159.02*** | 168.94*** |
|  | *H****^2^*** | 0.68 | 0.53 | 0.97 | 0.95 | 0.80 | 0.76 | 0.91 | 0.91 | 0.78 | 0.74 | 0.83 | 0.75 | 0.65 | 0.58 | 0.83 | 0.89 | 0.84 | 0.74 | 0.77 | 0.73 | 0.79 | 0.79 | 0.85 | 0.83 |
|  | *σ****^2^_G×T_*** | < 0.01^ns^ | | 0.22^ns^ | | 0.02^ns^ | | 0.02** | | 0.76^ns^ | | 0.03** | | < 0.01^ns^ | | < 0.01^ns^ | | 1.52* | | 2.18* | | 8.03* | | 6.54^ns^ | |
| CAMP (n = 11) | max | 4.54 | 4.57 | 95.04 | 98.73 | 4.77 | 5.54 | 0.87 | 1.04 | 26.70 | 27.44 | 3.00 | 2.49 | 9.54 | 7.00 | 81.97 | 82.46 | 30.70 | 31.06 | 38.17 | 39.89 | 92.66 | 92.43 | 138.51 | 148.31 |
|  | mean | 3.07 ± 0.93a | 3.18 ± 0.96a | 88.11 ± 4.82a | 89.12 ± 5.45a | 3.92 ± 0.72a | 4.88 ± 0.84b | 0.33 ± 0.26a | 0.39 ± 0.33a | 21.13 ± 3.65a | 22.28 ± 2.89a | 1.91 ± 0.66a | 1.68 ± 0.55a | 4.11 ± 3.04a | 3.59 ± 2.33a | 73.44 ± 5.86a | 75.37 ± 5.39a | 25.56 ± 3.31a | 24.83 ± 3.46a | 31.34 ± 5.16a | 34.03 ± 3.71a | 73.61 ± 15.05a | 76.79 ± 11.04a | 110 ± 17.01a | 115.99 ± 15.37a |
|  | min | 1.32 | 1.64 | 78.80 | 78.26 | 2.43 | 3.38 | 0.04 | 0.12 | 12.82 | 16.58 | 1.00 | 1.00 | -0.04 | -0.50 | 58.94 | 63.70 | 18.30 | 19.09 | 21.88 | 27.79 | 47.87 | 55.83 | 86.12 | 97.39 |
|  | *σ****^2^_G_*** | 0.55^ns^ | 0.22** | 22.21*** | 28.87*** | 0.38*** | 0.54*** | 0.02^ns^ | 0.07* | 11.47*** | 4.11* | 0.37*** | 0.22*** | 7.80*** | 4.44*** | 27.09*** | 24.99*** | 7.96*** | 7.04*** | 17.58*** | 6.83^ns^ | 192.56*** | 91.09*** | 240.57*** | 191.69*** |
|  | *H****^2^*** | 0.72 | 0.30 | 0.98 | 0.98 | 0.75 | 0.78 | 0.36 | 0.63 | 0.85 | 0.56 | 0.87 | 0.78 | 0.86 | 0.83 | 0.85 | 0.91 | 0.77 | 0.66 | 0.75 | 0.56 | 0.90 | 0.81 | 0.89 | 0.85 |
|  | *σ****^2^_G×T_*** | < 0.01^ns^ | | 1.23* | | 0.06^ns^ | | < 0.01^ns^ | | 0.64^ns^ | | 0.03^ns^ | | 0.45^ns^ | | 7.13* | | 3.95* | | < 0.01^ns^ | | < 0.01^ns^ | | < 0.01^ns^ | |
| GELB (n = 32) | max | 6.53 | 8.05 | 102.13 | 102.21 | 7.29 | 7.54 | 1.25 | 1.26 | 32.41 | 36.23 | 3.00 | 3.00 | 16.55 | 16.50 | 81.42 | 81.25 | 41.85 | 41.68 | 51.58 | 53.03 | 126.20 | 122.71 | 162.59 | 157.60 |
|  | mean | 4.08 ± 1.06a | 5.31 ± 1.79b | 92.50 ± 4.70a | 92.53 ± 4.53a | 5.45 ± 1.06a | 6.32 ± 0.94b | 0.31 ± 0.28a | 0.37 ± 0.29a | 26.73 ± 3.70a | 28.78 ± 4.49a | 1.92 ± 0.61a | 1.56 ± 0.62b | 6.67 ± 3.71a | 5.58 ± 3.67a | 72.43 ± 5.99a | 74.17 ± 4.85a | 32.54 ± 5.00a | 33.14 ± 4.36a | 42.47 ± 6.14a | 43.17 ± 5.97a | 90.54 ± 13.91a | 91.20 ± 15.68a | 124.28 ± 18.73a | 124.69 ± 17.29a |
|  | min | 1.85 | 1.72 | 83.40 | 83.06 | 2.83 | 3.43 | -0.03 | 0.02 | 19.68 | 19.26 | 1.00 | 0.99 | 1.29 | -1.50 | 52.95 | 60.79 | 23.76 | 24.53 | 28.01 | 28.66 | 55.93 | 56.70 | 89.62 | 81.16 |
|  | *σ****^2^_G_*** | 0.88*** | 2.68*** | 21.10*** | 19.65*** | 0.96*** | 0.71*** | 0.03ns | 0.04*** | 10.66*** | 16.06*** | 0.32*** | 0.31*** | 12.26*** | 13.06*** | 27.82*** | 21.15*** | 27.36*** | 16.69*** | 43.22*** | 30.18*** | 253.09*** | 366.55*** | 355.57*** | 267.80*** |
|  | *H****^2^*** | 0.81 | 0.84 | 0.97 | 0.96 | 0.88 | 0.83 | 0.42 | 0.51 | 0.84 | 0.83 | 0.85 | 0.84 | 0.91 | 0.93 | 0.85 | 0.89 | 0.92 | 0.82 | 0.88 | 0.85 | 0.92 | 0.94 | 0.93 | 0.89 |
|  | *σ****^2^_G×T_*** | 0.35** | | 1.04*** | | 0.09* | | < 0.01^ns^ | | 3.10** | | 0.05** | | < 0.01^ns^ | | 12.29*** | | 1.98* | | 2.32^ns^ | | 4.94^ns^ | | 8.35^ns^ | |
| SATU (n = 53) | max | 7.66 | 5.81 | 110.74 | 107.24 | 6.41 | 6.51 | 1.94 | 1.63 | 26.27 | 29.05 | 3.00 | 3.00 | 17.91 | 21.00 | 82.49 | 80.18 | 31.71 | 33.25 | 42.80 | 41.46 | 98.21 | 105.74 | 140.27 | 146.07 |
|  | mean | 2.42 ± 1.20a | 2.81 ± 1.04a | 95.30 ± 4.85a | 96.57 ± 4.26a | 3.19 ± 0.99a | 4.09 ± 1.02b | 0.46 ± 0.49a | 0.49 ± 0.48a | 17.79 ± 2.76a | 18.61 ± 2.87a | 2.12 ± 0.63a | 1.69 ± 0.50b | 7.26 ± 4.36a | 6.37 ± 4.66a | 70.74 ± 5.68a | 72.29 ± 4.38a | 20.35 ± 3.44a | 22.34 ± 3.27b | 26.02 ± 5.36a | 29.07 ± 4.09b | 61.61 ± 14.99a | 65.13 ± 12.73a | 103.05 ± 18.76a | 106.45 ± 19.28a |
|  | min | 0.73 | 1.02 | 84.71 | 86.18 | 1.67 | 2.04 | -0.02 | -0.01 | 13.37 | 12.95 | 1.00 | 0.99 | 1.11 | 0.50 | 59.34 | 62.71 | 14.88 | 16.05 | 14.02 | 19.21 | 29.90 | 41.20 | 58.04 | 56.87 |
|  | *σ****^2^_G_*** | 1.11*** | 0.55*** | 22.68*** | 17.26*** | 0.81*** | 0.87*** | 0.20*** | 0.19*** | 5.19*** | 4.62*** | 0.34*** | 0.19*** | 17.32*** | 20.71*** | 28.02*** | 17.88*** | 9.25*** | 6.37*** | 21.25*** | 9.92*** | 198.90*** | 134.14*** | 319.16*** | 333.85*** |
|  | *H****^2^*** | 0.84 | 0.52 | 0.98 | 0.96 | 0.87 | 0.85 | 0.83 | 0.83 | 0.72 | 0.59 | 0.86 | 0.75 | 0.93 | 0.96 | 0.86 | 0.88 | 0.80 | 0.64 | 0.78 | 0.64 | 0.90 | 0.86 | 0.92 | 0.91 |
|  | *σ****^2^_G×T_*** | < 0.01^ns^ | | 2.57*** | | 0.01^ns^ | | < 0.01^ns^ | | < 0.01^ns^ | | 0.02* | | 2.92*** | | 3.49** | | < 0.01^ns^ | | 0.95^ns^ | | 4.75^ns^ | | 23.10** | |
| STGA (n = 14) | max | 6.78 | 9.82 | 92.26 | 91.89 | 6.93 | 8.05 | 0.96 | 0.93 | 35.17 | 40.08 | 3.00 | 1.98 | 7.43 | 7.50 | 81.63 | 80.60 | 42.56 | 43.88 | 53.53 | 56.16 | 104.08 | 107.80 | 115.21 | 114.78 |
|  | mean | 4.83 ± 1.11a | 6.80 ± 2.07b | 86.84 ± 3.20a | 86.43 ± 3.55a | 5.85 ± 0.80a | 6.94 ± 0.66b | 0.40 ± 0.25a | 0.35 ± 0.21a | 27.17 ± 3.83a | 31.14 ± 5.85b | 1.36 ± 0.60a | 1.11 ± 0.28a | 4.30 ± 1.63a | 4.25 ± 2.20a | 79.20 ± 1.22a | 78.64 ± 1.26a | 32.99 ± 4.59a | 34.76 ± 4.44a | 41.86 ± 5.38a | 45.98 ± 6.05a | 86.88 ± 10.68a | 90.46 ± 8.84a | 99.18 ± 10.56a | 100.93 ± 11.47a |
|  | min | 3.15 | 3.43 | 81.25 | 79.69 | 4.27 | 6.07 | 0.09 | 0.12 | 23.19 | 22.03 | 1.00 | 0.98 | 0.76 | 1.00 | 77.34 | 76.34 | 23.57 | 27.85 | 33.34 | 37.76 | 67.18 | 79.40 | 76.27 | 79.36 |
|  | *σ****^2^_G_*** | 1.00*** | 3.91*** | 9.48*** | 11.97*** | 0.48*** | 0.26*** | 0.01^ns^ | < 0.01^ns^ | 12.74*** | 30.22*** | 0.36*** | 0.01^ns^ | 1.29^ns^ | 3.75*** | < 0.01^ns^ | < 0.01^ns^ | 18.48*** | 15.27*** | 22.31*** | 30.75*** | 78.44*** | 49.07*** | 72.53*** | 95.53*** |
|  | *H****^2^*** | 0.83 | 0.89 | 0.94 | 0.94 | 0.79 | 0.64 | 0.13 | 0.11 | 0.86 | 0.90 | 0.87 | 0.19 | 0.50 | 0.80 | 0.00 | 0.00 | 0.89 | 0.81 | 0.79 | 0.85 | 0.78 | 0.69 | 0.72 | 0.74 |
|  | *σ****^2^_G×T_*** | 1.29*** | | 0.99* | | < 0.01^ns^ | | < 0.01^ns^ | | 6.27*** | | 0.03^ns^ | | 1.50** | | < 0.01^ns^ | | 1.50^ns^ | | 2.45^ns^ | | 5.32^ns^ | | < 0.01^ns^ | |
| STRE (n = 30) | max | 6.13 | 9.02 | 102.50 | 103.55 | 7.04 | 7.95 | 1.01 | 1.39 | 32.72 | 38.07 | 3.00 | 2.99 | 10.16 | 8.50 | 80.74 | 81.61 | 38.59 | 43.18 | 47.65 | 50.90 | 102.73 | 108.89 | 142.91 | 138.87 |
|  | mean | 3.99 ± 1.13a | 5.09 ± 2.18b | 93.33 ± 4.02a | 93.03 ± 3.74a | 4.99 ± 1.16a | 6.17 ± 1.13b | 0.38 ± 0.26a | 0.36 ± 0.31a | 24.21 ± 4.14a | 27.27 ± 5.85b | 2.22 ± 0.49a | 1.78 ± 0.50b | 5.48 ± 2.59a | 4.12 ± 2.34b | 73.53 ± 6.68a | 75.04 ± 4.81a | 29.05 ± 5.38a | 31.66 ± 4.87a | 36.96 ± 6.74a | 40.28 ± 6.06b | 82.11 ± 10.24a | 87.13 ± 11.42a | 114.00 ± 13.49a | 113.05 ± 14.84a |
|  | min | 1.38 | 1.74 | 85.00 | 85.58 | 2.77 | 3.05 | 0.02 | 0.07 | 17.98 | 17.85 | 1.50 | 0.99 | -0.09 | -2.00 | 52.30 | 61.14 | 17.95 | 21.95 | 25.33 | 27.70 | 61.37 | 62.62 | 81.26 | 75.19 |
|  | *σ****^2^_G_*** | 1.01*** | 4.14*** | 15.18*** | 13.06*** | 1.20*** | 1.12*** | 0.01ns | 0.05*** | 14.32*** | 30.04*** | 0.18*** | 0.19*** | 5.25*** | 4.46*** | 30.16*** | 13.91*** | 26.40*** | 19.88*** | 38.65*** | 29.98*** | 77.00*** | 105.28*** | 147.01*** | 182.39*** |
|  | *H****^2^*** | 0.83 | 0.89 | 0.96 | 0.95 | 0.91 | 0.88 | 0.19 | 0.58 | 0.88 | 0.90 | 0.77 | 0.75 | 0.80 | 0.83 | 0.86 | 0.85 | 0.92 | 0.85 | 0.87 | 0.85 | 0.78 | 0.83 | 0.84 | 0.85 |
|  | *σ****^2^_G×T_*** | 0.49** | | 1.79*** | | 0.13* | | < 0.01^ns^ | | 1.99* | | 0.04^ns^ | | 1.33** | | 4.24** | | 1.99* | | 3.81^ns^ | | < 0.01^ns^ | | 4.23^ns^ | |
| WALL (n = 59) | max | 6.88 | 8.70 | 110.11 | 108.14 | 6.26 | 8.02 | 1.29 | 1.67 | 30.04 | 32.87 | 3.00 | 3.01 | 16.54 | 15.00 | 82.00 | 80.82 | 38.88 | 42.12 | 49.96 | 51.94 | 115.94 | 110.97 | 151.25 | 152.67 |
|  | mean | 3.12 ± 1.27a | 3.96 ± 1.71b | 94.76 ± 5.14a | 95.23 ± 4.72a | 4.09 ± 1.20a | 5.23 ± 1.19b | 0.33 ± 0.31a | 0.33 ± 0.33a | 22.43 ± 3.41a | 24.62 ± 3.60b | 2.20 ± 0.62a | 1.65 ± 0.55b | 8.11 ± 3.78a | 6.40 ± 3.30b | 69.50 ± 8.03a | 71.94 ± 6.49a | 27.04 ± 4.71a | 29.18 ± 5.08b | 34.62 ± 6.55a | 38.37 ± 6.40b | 76.97 ± 15.14a | 82.44 ± 12.67b | 111.93 ± 14.80a | 111.94 ± 15.84a |
|  | min | 0.97 | 0.98 | 81.73 | 82.28 | 1.46 | 2.41 | 0.00 | 0.02 | 15.83 | 16.18 | 1.00 | 0.98 | 1.41 | 1.50 | 34.69 | 42.46 | 16.86 | 18.75 | 18.42 | 24.08 | 41.97 | 51.36 | 82.95 | 81.89 |
|  | *σ****^2^_G_*** | 1.36*** | 2.36*** | 25.56*** | 21.41*** | 1.28*** | 1.26*** | 0.05*** | 0.08*** | 9.19*** | 9.48*** | 0.33*** | 0.24*** | 12.86*** | 9.87*** | 65.71*** | 20.49*** | 19.80*** | 21.80*** | 36.78*** | 34.32*** | 205.15*** | 133.13*** | 186.82*** | 212.67*** |
|  | *H****^2^*** | 0.87 | 0.82 | 0.98 | 0.97 | 0.91 | 0.89 | 0.55 | 0.66 | 0.82 | 0.75 | 0.86 | 0.79 | 0.91 | 0.91 | 0.93 | 0.89 | 0.89 | 0.86 | 0.86 | 0.86 | 0.90 | 0.86 | 0.87 | 0.86 |
|  | *σ****^2^_G×T_*** | 0.06^ns^ | | 0.59** | | 0.03^ns^ | | < 0.01^ns^ | | < 0.01^ns^ | | 0.06*** | | 2.38*** | | 8.91*** | | 1.91* | | 5.87*** | | 10.18* | | < 0.01^ns^ | |

**Table S3** Population-specific summary statistics for the evaluated phenotypic traits (denoted by "-" for -P and "+" for +P treatment) measured in EWE20. $\sigma_{G}^{2}$ denotes the genotypic variance, $H^{2}$ the heritability and $\sigma_{G\times T}^{2}$ the genotype-by-treatment interaction variance. The minimum values are abbreviated by 'min'. the maximum values by 'max' and the mean values with the standard deviation by 'mean **±** sd'. Means with shared letters are not significantly different (at p < 0.05. Tukey-test) in performance across treatments. Asterisks display significance of variance components as ns, > 0.05; *, 0.01 < p ≤ 0.05; **, 0.001 < p ≤ 0.01; ***, p ≤ 0.001. Populations are abbreviated as follows: Elite Dent (ED). Elite Flint (EF). Campan Galade (CG). Gelber Badischer Landmais (GB). Satu Mare (SM). St Galler Rheintaler (RT). Strenzfelder (SF) and Walliser (WA).

|  |  | - BM1 | + BM1 | - DTA | + DTA | - EV | + EV | - Gyield | + Gyield | - PH1 | + PH1 | - ASI | + ASI | - GDM | + GDM | - PH2 | + PH2 | - PH3 | + PH3 | - PHF | + PHF |
| --- | --- | --- | --- | --- | --- | --- | --- | --- | --- | --- | --- | --- | --- | --- | --- | --- | --- | --- | --- | --- | --- |
| ED  (n = 100) | max | 15.21 | 17.01 | 101.06 | 101.91 | 8.07 | 9.09 | 6.37 | 6.43 | 23.43 | 23.23 | 6.02 | 5.50 | 85.74 | 86.99 | 34.93 | 35.69 | 60.36 | 56.97 | 228.08 | 224.32 |
|  | mean | 9.88 ± 2.08a | 9.25 ± 2.50a | 88.33 ± 3.73a | 89.11 ± 3.87a | 6.83 ± 0.58a | 6.27 ± 0.76b | 3.77 ± 1.27a | 3.95 ± 1.20a | 18.30 ± 1.78a | 18.23 ± 2.04a | 0.35 ± 1.46a | 0.35 ± 1.65a | 77.06 ± 5.27a | 80.13 ± 4.73b | 26.55 ± 2.95a | 27.07 ± 3.68a | 45.01 ± 5.33a | 43.71 ± 5.52a | 183.12 ± 17.91a | 179.21 ± 18.53a |
|  | min | 4.78 | 4.42 | 79.27 | 81.20 | 4.57 | 4.23 | 0.89 | 1.12 | 13.97 | 13.17 | -4.03 | -4.00 | 59.77 | 65.53 | 19.58 | 19.30 | 28.69 | 30.89 | 133.10 | 132.64 |
|  | *σ****^2^_G_*** | 3.05*** | 3.98*** | 13.02*** | 14.16*** | 0.24*** | 0.38*** | 1.51*** | 1.37*** | 2.26*** | 2.86*** | 1.15*** | 1.89*** | 26.32*** | 21.56*** | 6.04*** | 9.83*** | 20.86*** | 21.95*** | 284.51*** | 304.13*** |
|  | *H****^2^*** | 0.71 | 0.73 | 0.94 | 0.95 | 0.76 | 0.75 | 0.94 | 0.95 | 0.72 | 0.76 | 0.54 | 0.70 | 0.96 | 0.96 | 0.74 | 0.79 | 0.77 | 0.82 | 0.90 | 0.90 |
|  | *σ****^2^_G×T_*** | 0.30^ns^ | | 0.02^ns^ | | 0.02^ns^ | | 0.04** | | < 0.01^ns^ | | < 0.01^ns^ | | 0.46* | | 0.24^ns^ | | < 0.01^ns^ | | < 0.01^ns^ | |
| EF (n = 100) | max | 18.05 | 16.09 | 92.85 | 93.70 | 8.57 | 8.91 | 4.59 | 4.69 | 25.53 | 25.25 | 5.54 | 5.50 | 87.07 | 87.72 | 36.08 | 36.55 | 64.97 | 58.99 | 212.51 | 212.62 |
|  | mean | 11.90 ± 2.19a | 11.50 ± 2.16a | 82.18 ± 3.54a | 82.96 ± 3.62a | 7.53 ± 0.55a | 7.31 ± 0.73a | 2.73 ± 0.90a | 2.97 ± 0.84b | 19.79 ± 1.80a | 20.03 ± 1.95a | 0.51 ± 1.40a | 0.52 ± 1.17a | 80.44 ± 4.25a | 83.43 ± 3.60b | 28.84 ± 2.80a | 30.58 ± 3.01b | 50.12 ± 4.44a | 49.85 ± 5.07a | 172.41 ± 16.27a | 169.64 ± 16.94a |
|  | min | 6.97 | 6.64 | 75.69 | 75.41 | 6.07 | 5.22 | 0.37 | 0.58 | 14.96 | 14.52 | -2.97 | -3.50 | 65.22 | 67.92 | 22.22 | 21.33 | 40.48 | 36.68 | 113.24 | 110.18 |
|  | *σ****^2^_G_*** | 3.03*** | 2.73*** | 11.63*** | 12.26*** | 0.21*** | 0.34*** | 0.70*** | 0.62*** | 2.19*** | 2.55*** | 0.96*** | 0.55*** | 16.45*** | 12.00*** | 4.96*** | 5.57*** | 11.51*** | 17.64*** | 226.03*** | 248.71*** |
|  | *H****^2^*** | 0.71 | 0.64 | 0.94 | 0.95 | 0.74 | 0.73 | 0.88 | 0.90 | 0.71 | 0.73 | 0.50 | 0.41 | 0.93 | 0.93 | 0.70 | 0.68 | 0.65 | 0.78 | 0.88 | 0.88 |
|  | *σ****^2^_G×T_*** | < 0.01^ns^ | | < 0.01^ns^ | | < 0.01^ns^ | | 0.05*** | | 0.07^ns^ | | < 0.01^ns^ | | 0.14^ns^ | | 0.16^ns^ | | < 0.01^ns^ | | < 0.01^ns^ | |
| CAMP (n = 11) | max | 14.48 | 12.60 | 89.38 | 90.89 | 7.80 | 7.28 | 1.88 | 1.91 | 20.36 | 22.74 | 5.00 | 3.00 | 86.46 | 87.08 | 29.85 | 34.15 | 51.87 | 53.40 | 179.75 | 184.41 |
|  | mean | 11.32 ± 1.82a | 10.10 ± 1.92a | 78.95 ± 4.89a | 80.07 ± 5.10a | 6.78 ± 0.61a | 6.35 ± 0.79a | 0.85 ± 0.49a | 0.87 ± 0.48a | 18.20 ± 1.75a | 18.67 ± 2.14a | 1.09 ± 1.50a | 0.77 ± 1.36a | 81.83 ± 4.58a | 83.94 ± 3.76a | 26.57 ± 2.56a | 28.56 ± 3.71a | 45.30 ± 4.22a | 44.67 ± 6.19a | 151.29 ± 14.99a | 149.87 ± 17.36a |
|  | min | 8.05 | 6.78 | 72.69 | 72.41 | 5.45 | 4.76 | 0.24 | 0.12 | 15.46 | 14.95 | -0.50 | -2.04 | 70.64 | 73.56 | 20.24 | 22.53 | 36.11 | 32.29 | 126.32 | 120.03 |
|  | *σ****^2^_G_*** | 1.96* | 1.94* | 23.32*** | 25.57*** | 0.30*** | 0.49*** | 0.14* | 0.15** | 2.24*** | 3.46*** | 1.24* | 0.56*** | 19.23*** | 12.94*** | 3.72*** | 10.14*** | 9.47* | 28.91*** | 182.64*** | 261.40*** |
|  | *H****^2^*** | 0.62 | 0.56 | 0.97 | 0.97 | 0.80 | 0.80 | 0.60 | 0.68 | 0.72 | 0.79 | 0.56 | 0.41 | 0.94 | 0.94 | 0.64 | 0.80 | 0.60 | 0.86 | 0.86 | 0.88 |
|  | *σ****^2^_G×T_*** | < 0.01^ns^ | | < 0.01^ns^ | | < 0.01^ns^ | | < 0.01^ns^ | | 0.07^ns^ | | < 0.01^ns^ | | 1.39* | | 0.50^ns^ | | < 0.01^ns^ | | < 0.01^ns^ | |
| GELB (n = 32) | max | 26.26 | 16.68 | 89.81 | 93.70 | 9.21 | 8.45 | 2.44 | 3.15 | 26.17 | 25.99 | 8.50 | 7.50 | 86.65 | 88.85 | 39.53 | 43.64 | 67.21 | 64.97 | 213.43 | 207.50 |
|  | mean | 12.62 ± 3.81a | 12.37 ± 2.72a | 83.14 ± 3.39a | 84.11 ± 3.85a | 7.63 ± 0.68a | 7.29 ± 0.80a | 0.88 ± 0.67a | 1.16 ± 0.72a | 21.02 ± 2.21a | 21.71 ± 2.19a | 1.75 ± 2.46a | 2.11 ± 2.84a | 81.48 ± 3.10a | 83.93 ± 2.76b | 31.54 ± 3.44a | 34.65 ± 4.45b | 54.42 ± 6.09a | 54.75 ± 6.89a | 174.89 ± 20.96a | 171.29 ± 20.24a |
|  | min | 5.82 | 6.71 | 76.38 | 76.82 | 5.71 | 5.81 | 0.06 | 0.06 | 17.59 | 17.33 | -3.96 | -4.00 | 74.59 | 75.52 | 22.69 | 26.22 | 43.11 | 40.06 | 126.58 | 112.49 |
|  | *σ****^2^_G_*** | 10.00*** | 5.11*** | 10.60*** | 14.11*** | 0.37*** | 0.44*** | 0.34*** | 0.44*** | 3.95*** | 3.52*** | 5.07*** | 7.18*** | 8.12*** | 6.68*** | 10.19*** | 16.84*** | 48.89*** | 43.93*** | 677.96*** | 606.47*** |
|  | *H****^2^*** | 0.89 | 0.77 | 0.93 | 0.95 | 0.83 | 0.78 | 0.79 | 0.86 | 0.82 | 0.79 | 0.84 | 0.90 | 0.87 | 0.88 | 0.83 | 0.87 | 0.89 | 0.90 | 0.96 | 0.95 |
|  | *σ****^2^_G×T_*** | 0.85^ns^ | | 1.10*** | | < 0.01^ns^ | | 0.07*** | | 0.76* | | 1.34*** | | 0.13^ns^ | | 2.59** | | 4.95* | | 15.92^ns^ | |
| SATU (n = 53) | max | 16.19 | 14.97 | 92.54 | 90.91 | 8.58 | 7.97 | 3.76 | 3.72 | 21.55 | 22.19 | 9.00 | 11.50 | 85.68 | 86.96 | 32.43 | 36.78 | 59.93 | 52.02 | 196.18 | 198.13 |
|  | mean | 8.91 ± 3.52a | 8.99 ± 2.33a | 83.51 ± 3.87a | 83.66 ± 3.31a | 5.94 ± 1.20a | 5.85 ± 0.82a | 1.20± 0.90a | 1.38 ± 0.81a | 16.69 ± 1.73a | 17.08 ± 1.95a | 2.66 ± 2.54a | 2.40 ± 2.31a | 76.62 ± 5.26a | 79.39 ± 5.36b | 23.96 ± 3.55a | 25.68 ± 3.65b | 39.60 ± 8.03a | 41.07 ± 5.13a | 146.79 ± 23.62a | 146.27 ± 18.77a |
|  | min | 1.88 | 4.23 | 74.79 | 75.30 | 1.97 | 2.95 | 0.02 | 0.15 | 13.47 | 12.85 | -0.52 | -1.00 | 64.69 | 63.83 | 15.80 | 16.71 | 20.70 | 27.81 | 91.92 | 97.90 |
|  | *σ****^2^_G_*** | 10.62*** | 3.80*** | 13.98*** | 10.11*** | 1.34*** | 0.50*** | 0.71*** | 0.58*** | 2.03*** | 2.71*** | 5.48*** | 4.51*** | 26.01*** | 28.87*** | 9.58*** | 5.57*** | 54.08*** | 20.08*** | 513.43*** | 316.39*** |
|  | *H****^2^*** | 0.90 | 0.72 | 0.95 | 0.94 | 0.95 | 0.80 | 0.89 | 0.89 | 0.70 | 0.75 | 0.85 | 0.85 | 0.96 | 0.97 | 0.82 | 0.80 | 0.90 | 0.80 | 0.94 | 0.90 |
|  | *σ****^2^_G×T_*** | 1.64*** | | 1.23*** | | 0.21*** | | < 0.01^ns^ | | < 0.01^ns^ | | 0.40* | | 1.57*** | | 1.03^ns^ | | 8.63*** | | 46.12*** | |
| STGA (n = 14) | max | 18.61 | 17.72 | 83.14 | 83.70 | 8.95 | 8.99 | 2.41 | 2.29 | 25.61 | 25.95 | 3.97 | 3.50 | 86.12 | 87.29 | 38.44 | 43.90 | 68.05 | 61.87 | 172.71 | 167.77 |
|  | mean | 13.98 ± 3.04a | 12.94 ± 2.25a | 78.71 ± 2.50a | 79.88 ± 2.66a | 7.96 ± 0.58a | 7.53 ± 0.83a | 0.95 ± 0.52a | 0.96 ± 0.49a | 22.25 ± 2.51a | 22.73 ± 2.71a | 1.90 ± 1.42a | 1.71 ± 1.16a | 82.64 ± 2.17a | 85.40 ± 1.35b | 32.69 ± 3.53a | 36.20 ± 4.25b | 53.10 ± 6.71a | 53.03 ± 5.69a | 150.87 ± 16.93a | 143.12 ± 16.52a |
|  | min | 7.96 | 8.54 | 75.10 | 76.23 | 6.87 | 6.38 | 0.26 | 0.35 | 18.11 | 17.74 | -0.51 | 0.00 | 78.04 | 82.21 | 27.06 | 29.29 | 42.33 | 44.48 | 116.18 | 107.76 |
|  | *σ****^2^_G_*** | 7.87** | 3.35*** | 5.14*** | 6.29*** | 0.28*** | 0.53*** | 0.18** | 0.15*** | 5.62*** | 6.13*** | 1.00* | 0.50*** | 3.31*** | 0.83^ns^ | 10.67*** | 14.22*** | 39.67*** | 27.08*** | 252.99*** | 230.58*** |
|  | *H****^2^*** | 0.87 | 0.69 | 0.87 | 0.90 | 0.79 | 0.81 | 0.65 | 0.68 | 0.87 | 0.87 | 0.51 | 0.39 | 0.73 | 0.49 | 0.83 | 0.85 | 0.86 | 0.85 | 0.89 | 0.87 |
|  | *σ****^2^_G×T_*** | 0.34^ns^ | | < 0.01^ns^ | | 0.07^ns^ | | < 0.01^ns^ | | 0.61^ns^ | | < 0.01^ns^ | | < 0.01^ns^ | | 0.85^ns^ | | 5.73^ns^ | | 19.93^ns^ | |
| STRE (n = 30) | max | 16.65 | 18.41 | 95.36 | 94.34 | 8.72 | 8.53 | 3.71 | 2.24 | 23.47 | 25.74 | 10.06 | 11.50 | 86.73 | 87.28 | 38.21 | 39.53 | 63.96 | 62.19 | 182.32 | 179.12 |
|  | mean | 11.98 ± 3.00a | 11.81 ± 3.09a | 83.39 ± 4.29a | 84.30 ± 4.34a | 7.38 ± 0.84a | 6.97 ± 0.91a | 1.12 ± 0.84a | 1.01 ± 0.60a | 20.00 ± 2.07a | 19.75 ± 2.56a | 1.66 ± 3.36a | 1.34 ± 3.70a | 82.62 ± 3.83a | 84.99 ± 3.68b | 29.97 ± 3.42a | 32.03 ± 4.81a | 50.47 ± 7.27a | 50.25 ± 6.62a | 161.27 ± 19.87a | 154.80 ± 18.66a |
|  | min | 5.22 | 6.22 | 77.41 | 77.09 | 5.56 | 5.13 | -0.02 | 0.04 | 14.42 | 14.29 | -6.96 | -9.04 | 66.83 | 67.67 | 22.09 | 22.28 | 32.49 | 38.68 | 100.23 | 100.30 |
|  | *σ****^2^_G_*** | 7.45*** | 7.39*** | 17.22*** | 17.83*** | 0.60*** | 0.65*** | 0.59*** | 0.28*** | 3.12*** | 5.45*** | 9.64*** | 12.18*** | 13.15*** | 12.65*** | 8.45*** | 19.06*** | 43.59*** | 34.67*** | 349.70*** | 311.97*** |
|  | *H****^2^*** | 0.86 | 0.83 | 0.96 | 0.96 | 0.89 | 0.84 | 0.87 | 0.79 | 0.78 | 0.86 | 0.91 | 0.94 | 0.92 | 0.94 | 0.80 | 0.88 | 0.88 | 0.88 | 0.92 | 0.90 |
|  | *σ****^2^_G×T_*** | 0.20^ns^ | | 0.79** | | 0.01^ns^ | | < 0.01^ns^ | | 0.89** | | 0.20^ns^ | | 0.04^ns^ | | 0.61^ns^ | | 4.06* | | < 0.01^ns^ | |
| WALL (n = 59) | max | 20.70 | 19.54 | 96.84 | 98.48 | 9.04 | 8.85 | 2.45 | 2.49 | 26.12 | 24.05 | 7.50 | 9.50 | 86.12 | 87.10 | 36.08 | 39.49 | 65.48 | 69.28 | 193.76 | 192.58 |
|  | mean | 11.27 ± 2.83a | 10.81 ± 2.99a | 83.38 ± 4.37a | 84.22 ± 4.01a | 7.43 ± 0.80a | 7.03 ± 0.83b | 0.98 ± 0.58a | 1.10 ± 0.58a | 19.69 ± 2.30a | 19.80 ± 2.23a | 3.00 ± 2.17a | 2.97 ± 2.30a | 78.93 ± 4.90a | 81.80 ± 4.76b | 29.66 ± 3.43a | 32.13 ± 4.13b | 50.99 ± 6.41a | 52.47 ± 6.22a | 161.45 ± 15.32a | 161.40 ± 12.68a |
|  | min | 5.68 | 4.30 | 73.06 | 77.33 | 5.53 | 5.44 | 0.07 | 0.19 | 14.45 | 15.30 | -2.50 | -1.00 | 63.02 | 63.55 | 20.65 | 23.65 | 36.85 | 36.12 | 127.96 | 136.82 |
|  | *σ****^2^_G_*** | 6.49*** | 6.64*** | 18.14*** | 15.21*** | 0.56*** | 0.52*** | 0.23*** | 0.26*** | 4.30*** | 3.88*** | 3.72*** | 4.43*** | 22.73*** | 21.75*** | 8.98*** | 13.70*** | 32.94*** | 30.98*** | 198.44*** | 121.30*** |
|  | *H****^2^*** | 0.84 | 0.82 | 0.96 | 0.96 | 0.88 | 0.81 | 0.72 | 0.78 | 0.83 | 0.81 | 0.79 | 0.85 | 0.95 | 0.96 | 0.81 | 0.84 | 0.84 | 0.86 | 0.87 | 0.78 |
|  | *σ****^2^_G×T_*** | 0.76* | | 0.61*** | | 0.03^ns^ | | < 0.01^ns^ | | 0.28^ns^ | | 0.83*** | | 0.28^ns^ | | 1.35* | | 4.67** | | < 0.01^ns^ | |

# **Supplementary Figures**

**Figure S1** Weather data of the three environments during the growing season.


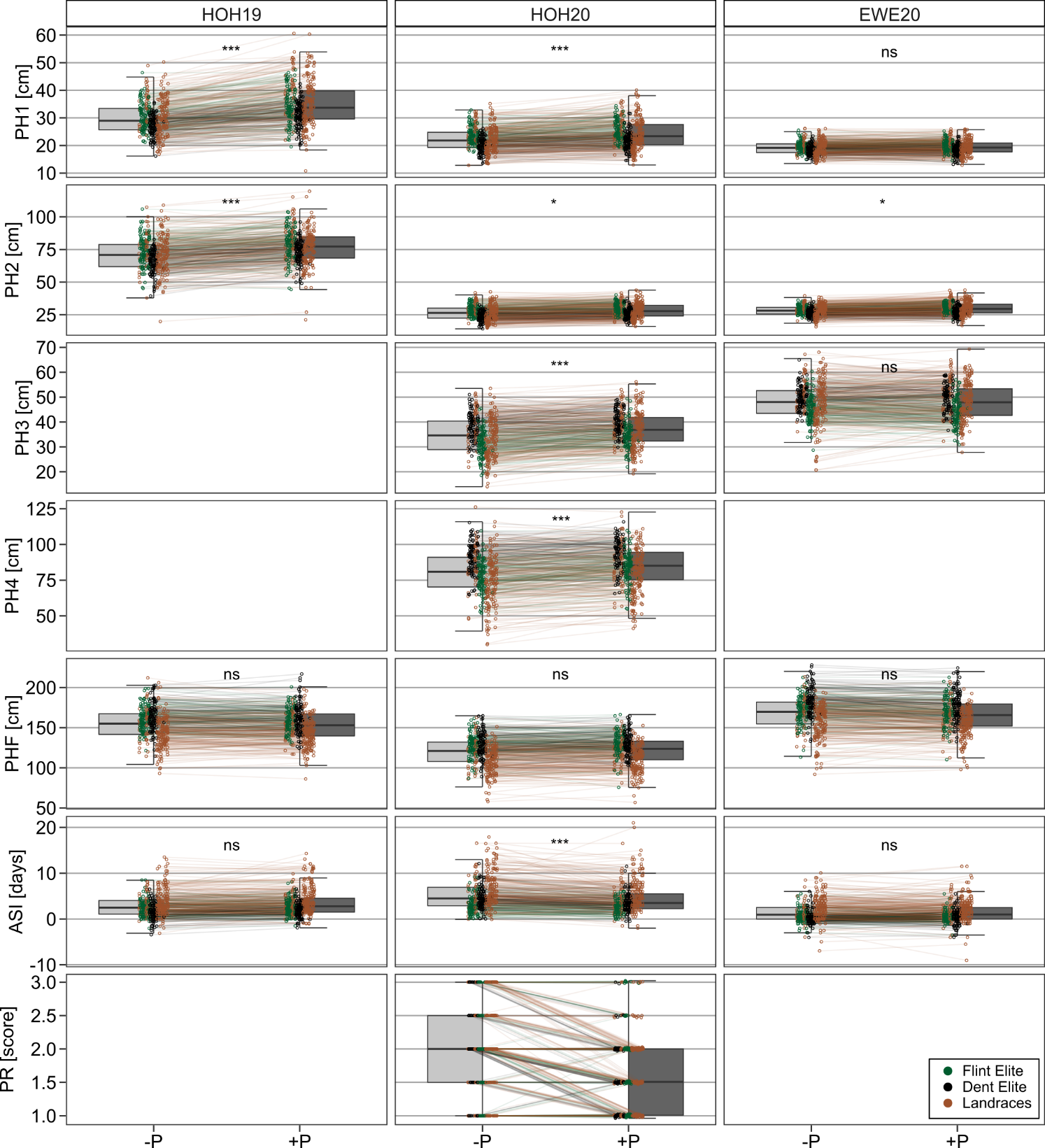


**Figure S2** Trait differences between P treatments. Boxplots show trait distributions with (+P) or without (-P) starter fertilizer for each environment (HOH19. HOH20. EWE20). Note that genotypes in each treatment are connected by lines and colored as either Dent Elite, Flint Elite or landrace doubled haploid lines. Asterisks show differences between P treatment: ns, p > 0.05; *, 0.01 < p ≤ 0.05; **, 0.001 < p ≤ 0.01; ***, p ≤ 0.001.


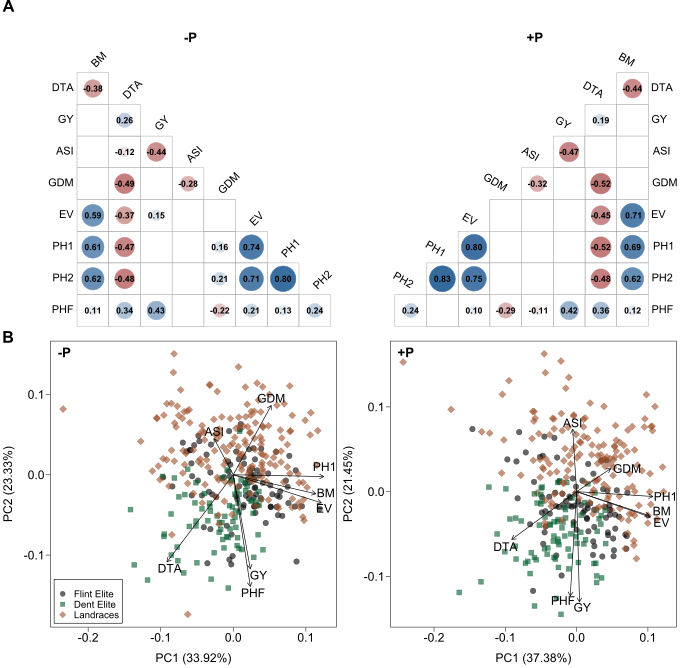


**Figure S3** Relationships among traits in HOH19. **(A)** Correlation matrixes and **(B)** principal component analysis of the lines based on phenotypic trait data. Results are shown without (-P) and with (+P) starter fertilizer. Blank fields in **(A)** are non-significant correlations. The genotypes in **(B)** are assigned to their material group as Dent Elite, Flint Elite and landrace doubled haploid lines.


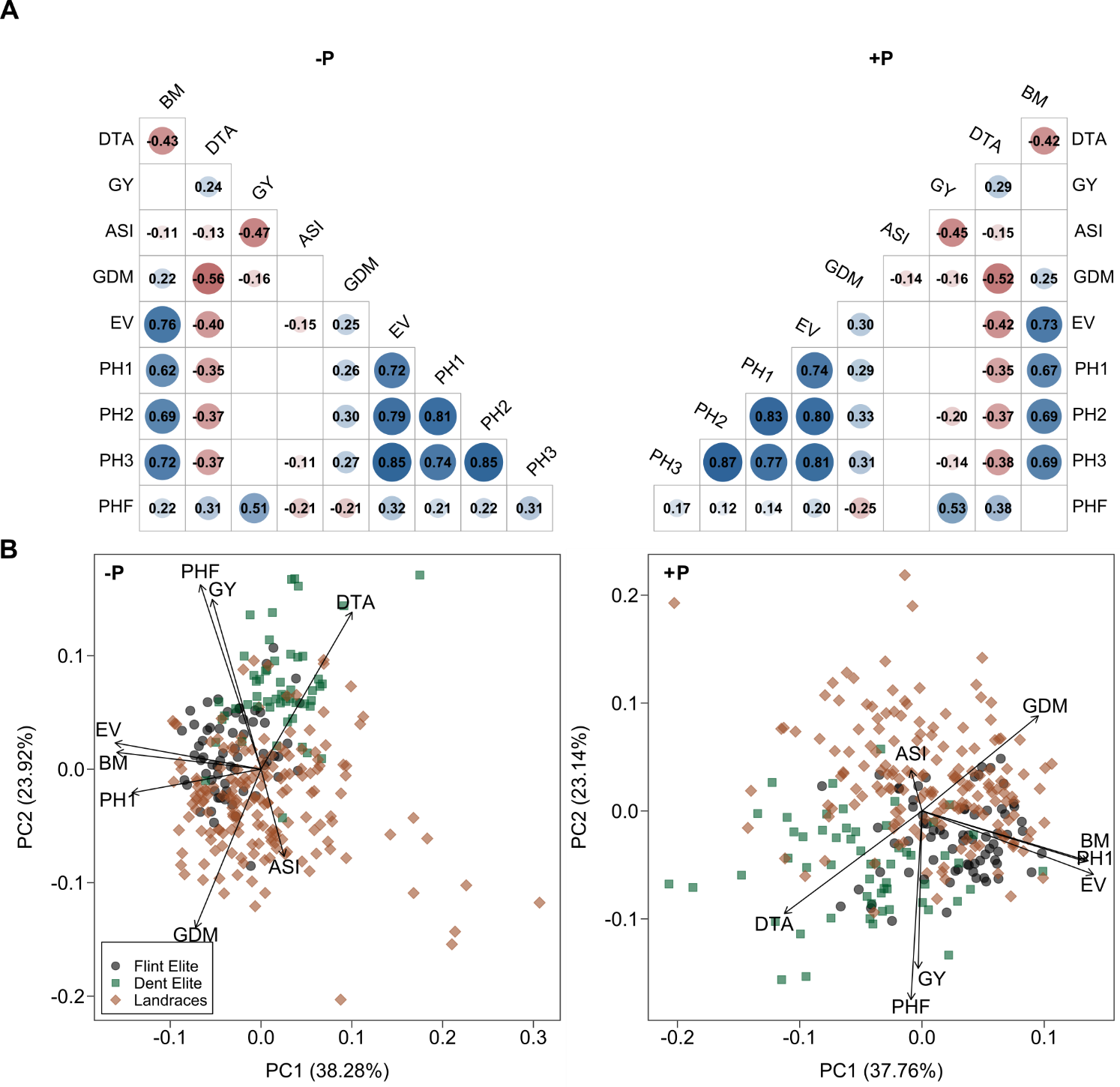


**Figure S4** Relationships among traits in EWE20. **(A)** Correlation matrixes and **(B)** principal component analysis of the lines based on phenotypic trait data. Results are shown without (-P) and with (+P) starter fertilizer. Blank fields in **(A)** are non-significant correlations. The genotypes in **(B)** are assigned to their material group as Dent Elite, Flint Elite and landrace doubled haploid lines.
